# Designing and conducting proof-of-concept chronic pain analgesic clinical trials

**DOI:** 10.1097/PR9.0000000000000697

**Published:** 2019-02-26

**Authors:** Claudia M. Campbell, Ian Gilron, Tina Doshi, Srinivasa Raja

**Affiliations:** a Department of Psychiatry and Behavioral Sciences, Johns Hopkins University, Baltimore, MD, USA; b Departments of Anesthesiology & Perioperative Medicine and Biomedical & Molecular Sciences, Queen's University, Kingston, ON, Canada; c Division of Pain Medicine, Johns Hopkins University, Department of Anesthesiology and Critical Care Medicine, Baltimore, MD, USA

**Keywords:** Pain, Proof-of-concept, Clinical trial, ACTTION, Quantitative sensory testing, Pain testing, Personalized medicine

## Abstract

**Introduction:**

The evolution of pain treatment is dependent on successful development and testing of interventions. Proof-of-concept (POC) studies bridge the gap between identification of a novel target and evaluation of the candidate intervention's efficacy within a pain model or the intended clinical pain population.

**Methods:**

This narrative review describes and evaluates clinical trial phases, specific POC pain trials, and approaches to patient profiling.

**Results:**

We describe common POC trial designs and their value and challenges, a mechanism-based approach, and statistical issues for consideration.

**Conclusion:**

Proof-of-concept trials provide initial evidence for target use in a specific population, the most appropriate dosing strategy, and duration of treatment. A significant goal in designing an informative and efficient POC study is to ensure that the study is safe and sufficiently sensitive to detect a preliminary efficacy signal (ie, a potentially valuable therapy). Proof-of-concept studies help avoid resources wasted on targets/molecules that are not likely to succeed. As such, the design of a successful POC trial requires careful consideration of the research objective, patient population, the particular intervention, and outcome(s) of interest. These trials provide the basis for future, larger-scale studies confirming efficacy, tolerability, side effects, and other associated risks.

## 1. Introduction

Advances in pain treatment depend on successfully transforming breakthroughs in basic research to new evidence-based treatment strategies. The journey from identifying a novel target to bringing a drug to the market, or development of a novel treatment approach, is intensive, extensive (10–15 years), and expensive (hundreds of millions of dollars). The path starts with basic science studies to identify a target, validate the biologic mechanisms of the target, and find a chemical that appropriately modifies the target. This is followed by preclinical studies in animal models to evaluate the drug's safety, efficacy, and potential toxicity. The final critical step includes clinical trials in humans to evaluate the candidate drug's safety and efficacy in a targeted patient population and confirmatory trials to obtain regulatory approval for its use (see Table 1 for a summary of different phases of clinical trials).^65,178^ Although the most common application of proof-of-concept (POC) studies in pain is for the testing of new drugs, these studies have also been used to identify pathophysiological mechanisms of pain in volunteers and individuals with select pain states and to validate new pain “models” and outcome measures in humans. Such studies enhance the pain research toolbox and can lead to new insights into the mechanisms and treatment of chronic pain. History suggests that this complex process is a risky endeavor because few new targets identified for pain therapy by preclinical research have led to successful treatments in clinical practice.

**Table 1:**
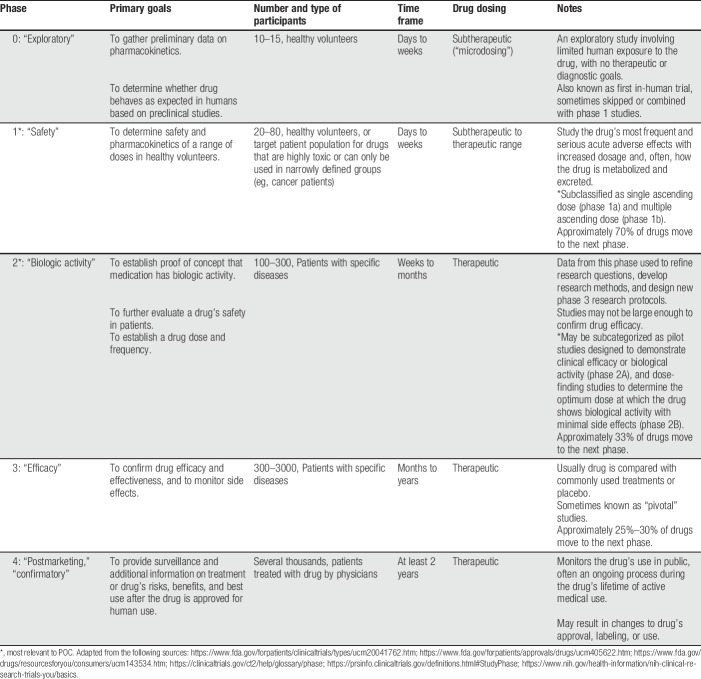
Classification of clinical trial phases.

A critical step in reducing risks during the translational process of advancing scientific discoveries into treatments is a well-constructed POC study.^63^ In contrast to phase 3 clinical trials that are aimed to evaluate a candidate treatment's benefit and safety profile in a specific patient population, POC studies are considered early-stage clinical trials performed to determine whether a treatment (eg, drug) interacts appropriately with its molecular target to achieve sufficient biological activity in humans. Proof-of-concept trials are usually designed to include fewer participants for a limited duration of follow-up and are an essential component of the development phase that helps decide whether to proceed to more comprehensive and expensive phase 3 clinical trials (“go/no-go” decision). They provide initial evidence for target use in a specific population, the most appropriate dosing strategy, and duration of treatment. A significant goal in designing an informative and efficient POC study is to ensure that the study is safe and sufficiently sensitive to detect a preliminary efficacy signal (ie, a potentially valuable therapy). Proof-of-concept studies help avoid resources being wasted on targets/molecules that are not likely to succeed. However, design of POC studies must have sufficient precision and assay sensitivity to ensure that a potentially successful treatment candidate is not inappropriately abandoned, eg, due to inconclusive results from a poorly designed trial.

## 2. Research questions for proof-of-concept trials

Proof-of-concepts generally provide the first opportunity to ask a research question in patients with various chronic pain conditions, and the nature of the research question will determine the selection of various trial design characteristics. Important differences from POC trials for acute pain treatments include clinical setting (eg, in-hospital postsurgical setting vs outpatient chronic pain clinic), patient population (eg, surgical patients vs patients with chronic disease), and duration of treatment (eg, hours to days vs weeks to months), and these differences have an important impact on various trial design features. From an analgesic drug development perspective, POC trials may be used to provide a preliminary evaluation of the safety and efficacy of a new molecular entity (eg, phase 2 trial) in a target chronic pain population.^63,65^ Proof-of-concept trials may also be useful to address a wide variety of other fundamental research goals, such as the development and validation of new pain-related outcome measures,^9^ elucidation of physiological pain mechanisms,^105^ identification of biomarkers to predict chronic pain treatment outcomes,^22,82,85^ assessing safety and preliminary efficacy of focused treatment strategies such as combination therapy,^67^ evaluating the utility of therapeutic drug monitoring for chronic pain management,^151^ and others.

### 2.1. Mechanistic studies

Proof-of-concept trials that randomize chronic pain patients to receive investigational or control interventions may be used to study a variety of different mechanisms of pain processing and/or analgesic treatment response. In support of a putative mechanism-specific response to analgesic treatment, a number of POC trials have evaluated treatment response according to pretrial pain phenotypes such as painful response to topical capsaicin^22^ or a “hypersensitivity phenotype” based on quantitative sensory testing (QST),^37^ suggestive of sensitized or irritable nociceptors. Some of these studies have demonstrated phenotype-specific differences in treatment response lending support to the concept of mechanism-based pain treatment.^5,150^ Other POC trials have made use of various techniques and biomarkers, such as functional brain imaging,^85^ microneurography,^147^ and genetic analysis,^159^ to understand interactions between mechanisms of pain processing and pain treatment outcomes.

### 2.2. Development of new analgesic treatment strategies

For the purposes of developing a new analgesic treatment, POC trials may use results from earlier phase 1 trials (eg, in healthy human volunteers) to guide the evaluation of safety, preliminary analgesic efficacy, and dose–response of a proposed new intervention in patients with chronic pain.^65^ For example, early phase 1 human trials of novel agents such as the glycine antagonist, GV196771,^86^ and the AMPA/kainate antagonist, LY293558,^143^ led to subsequent POC trials in peripheral neuropathic pain^170^ and migraine,^144^ respectively. As discussed later in this review, various trial features that are attractive at the POC stage are those that maximize the sensitivity and specificity for detecting analgesic efficacy in the investigational treatment. Further exploration of dose–response in terms of preliminary analgesic efficacy and adverse events may also be accomplished in POC trials beyond what was previously described in phase 1 trials.

The treatment comparator or control interventions, if any, should be carefully considered in POC trials and may include placebo, an alternative active comparator, or a lower dosage of the investigational treatment. Use of a placebo that is otherwise identically matched but devoid of specific biological effect is critical in quantifying the analgesic effects that are specific to the investigational treatment, that is, beyond any nonspecific effects that may be related to patients' treatment expectations, natural history of the pain condition, and/or regression to the mean (eg, patients with fluctuating pain levels may be more likely to enroll in a trial when pain levels are highest).^167^ Placebos are considered ethical as long as trial participants understand that they may withdraw from the trial at any time to pursue other pain treatment and/or that certain rescue analgesic treatment will be provided during the trial, so it is important to understand that these provisions may place limitations on analysis and interpretation of study findings. With respect to evaluating treatment safety in early POC trials, as well as in other types of trials, it should be recognized that participants treated with placebo might also report “nocebo” effects (ie, adverse symptoms or responses that may be attributable to negative expectations about the treatment or its side effects).^53^ The inclusion of an active comparator with previously proven efficacy in a POC study of an investigational treatment can serve to confirm “assay sensitivity” by demonstrating a statistically significant difference between the active comparator and placebo^113^ in situations where the study treatment fails to separate from placebo, thus failing to demonstrate efficacy. Aside from evaluating novel monotherapies, POC trials have also been useful to evaluate the added benefits of combining known treatments for chronic pain.^24,69^ In this regard, several POC trials have carefully compared analgesic combinations to their respective monotherapies,^66–68,70,84,97^ and several of these have guided subsequent, larger, industry-sponsored trials.^79,160^

## 3. Human experimental and clinical models of pain

A successful POC trial requires an appropriate patient population or model disease state. In clinical practice, chronic pain patients often present with a mixture of pain types, as well as psychosocial and cognitive factors, that defy easy classification and characterization of pain. Moreover, many of these patients have other medical conditions that may affect tolerability and response to potential treatments. As a result, inclusion of “typical” pain patients for small-scale clinical trials may make it difficult to demonstrate a true response to an experimental therapy. The use of experimental and clinical models of chronic pain allows for initial identification and definition of an appropriate target patient population (ie, with clearly defined characteristics) and clearer interpretation of experimental findings for specific populations, thus guiding future confirmatory trials.

### 3.1. Experimental models

Human experimental models of chronic pain have been used to test potential therapies through induction of reversible, experimentally induced pain. The ideal experimental pain model produces reversible or transient pain, does not cause long-term tissue damage or injury to the subject, is easy to perform, and provides reproducible results. The use of experimentally evoked pain models in healthy volunteers can be particularly useful when the target population is small, and it would be difficult to study an adequate number of patients, or when the safety of the therapy is not yet established and testing in patients with medical comorbidities would be inadvisable. Volunteer preclinical studies have a standardized intervention that minimizes variability of the injury stimulus across participants, tend to facilitate recruitment, are simpler to perform, and easier to replicate than patient studies. However, there is ongoing debate as to whether preclinical experimental models in humans are useful in predicting efficacy in patients with specific chronic pain conditions, especially since both the duration of pain and the test of drug treatment effects are brief.

One of the earliest and most commonly used preclinical models of pain is the burn injury model, in which cutaneous heat injury produces thermal and mechanical hyperalgesia in the area of the burn, as well as mechanical hyperalgesia in the surrounding area (Table 2).^138^ This model has been widely used to characterize the analgesic effects of numerous drugs, including morphine,^15^ lidocaine,^33^ ketamine,^87^ and ibuprofen^130^ in healthy participants. However, sensory changes produced by the burn injury model are known to diminish over the course of minutes to hours, and skin injury such as blistering and skin pigmentation changes may occur.^130^ Thus, investigators should be cautious when considering this method, and participants should be fully informed of the potential risks and consequences (as they should for all testing procedures). The brief thermal sensitization model also produces an area of thermal and mechanical hyperalgesia by delivering a 5-minute, 45°C stimulus to the thigh and measuring the area of hyperalgesia during the last 2 minutes of stimulation.^177^ This model is likely a better candidate in human studies because it is less likely to cause blistering and readily repeatable. In one report, brief thermal sensitization was performed twice daily for 5 consecutive days with no skin reactions^132^).

**Table 2:**
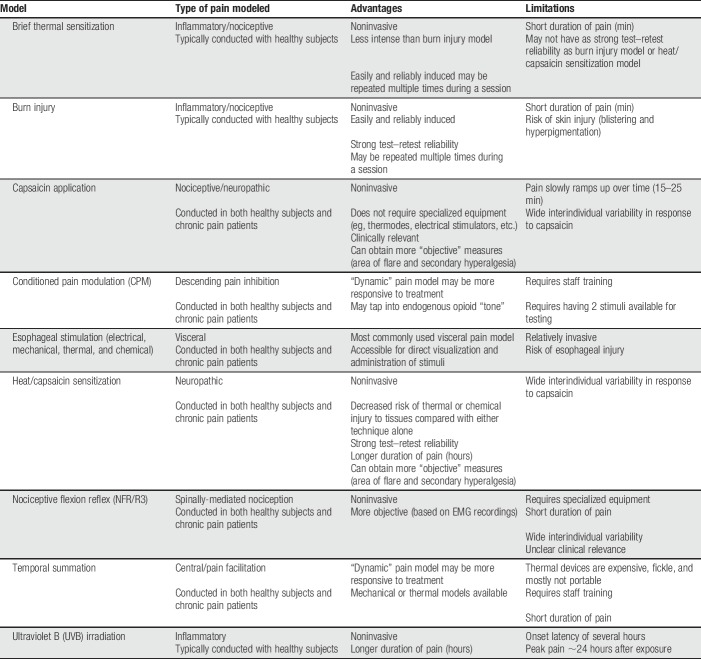
Common experimental pain models.

Another common experimental pain model involves the intradermal, intramuscular, or topical application of capsaicin.^149^ The administration of capsaicin results in acute, transient inflammatory pain the nature, duration, and intensity of which is dependent on route of administration and capsaicin concentration. Like thermal injury, the effects of capsaicin are often brief. The heat/capsaicin sensitization model combines topical capsaicin with thermal stimulation to prolong the ongoing pain and hyperalgesia.^23,133^ In an area of tissue pretreated with capsaicin, subsequent reapplications of heat can “rekindle” previous primary and secondary hyperalgesia, thereby extending experimental pain duration and intensity while reducing the potential for permanent injury from thermal or chemical burns.

The thermal and mechanical hyperalgesia evoked in the burn and capsaicin pain models, as well as in electrical stimulation techniques, are intended to correlate with features of neuropathic pain, although they also share features with nociceptive/inflammatory pain. Ultraviolet B (UVB) irradiation applied to small areas of skin produces stable mechanical and thermal hyperalgesia as a model of inflammatory pain.^10,78,158^ Visceral pain has been modeled in the esophagus using electrical, mechanical (dilation), thermal, and chemical (acid and capsaicin) stimulation, although the level of invasiveness and risk for esophageal perforation limit its use.^42,100,124^ Other less common modalities have also been used to model chronic pain states, including mechanical (pinch^109^), thermal (freeze lesion^108^ and laser^2^), chemical (acids and hypertonic saline^73,101^), and electrical stimulation,^101^ but their roles in translational pain research are not yet well-established.

Experimentally evoked pain models are useful for gathering additional data on safety and tolerability, dose finding, and as early explorations of potential analgesic efficacy or mechanism of action, particularly when a study needs to be completed within a relatively short time frame or has limited access to chronic pain patients. These models' ability to predict analgesic response has driven considerable interest in recent years^49,52,72,75,184^; however, it remains premature to suggest the effects of which drugs could be characterized by which testing methods. The selection of an appropriate model depends largely on the type of pain to be investigated and the hypothesized site of action of the treatment, but other considerations include the desired duration of elicited pain, the specific characteristics of the pain treatment to be studied, the resources available to the study team, and the expertise of the investigator. These same issues may also influence the implementation of the chosen experimental model, including the intensity, duration, and location of the pain-evoking stimuli.

When considering the use of experimental pain models, their potential limitations must also be taken into account. Although each of the models described above offers standardized protocols and reproducible results, large interindividual variability has been reported.^10,107,168^ Potential participants may therefore need to be screened to maximize the likelihood that they will respond as anticipated to the planned pain model. In addition, investigators should recognize that experimental pain models are susceptible to habituation, such that repeated applications of the same painful stimuli in the same participant may elicit less pain over time. Habituation may therefore limit both the duration and frequency of assessment of pain in experimental models.

Experimental pain models are not always the best choice for POC pain trials. For example, experimentally evoked pain does not always respond well to analgesics with established effectiveness, suggesting that these models are not perfectly correlated with specific chronic pain states.^168^ One possible explanation is that many pain treatments require repeated or prolonged administration of medication to detect benefit, and the nature of laboratory models does not allow for long-term assessment of therapy. Another major limitation of these models is that study participants typically do not have chronic pain, and acute injury may not accurately reflect the numerous physiologic and psychological changes that occur with chronic pain. In addition, although some experimental models create temporary central sensitization with a definable area of secondary hyperalgesia, researchers cannot ethically induce an actual, potentially long-term injury to a nerve. A localized pain model in volunteers is unlikely to simulate the multiple complex changes that are associated with a nerve injury in humans.

Consequently, findings in experimentally evoked pain studies may not readily translate to clinically relevant effects in actual chronic pain patient populations. Some researchers have advocated the use of brain imaging modalities to bridge this potential gap between experimental and clinical pain treatment efficacy, but more studies and refinement in techniques are necessary to explore the full potential of neuroimaging in the development of POC trials.^165^ As recently reviewed,^155^ sensory testing and imaging hold “promise as pain biomarkers and should be carefully considered for possible inclusion when designing clinical trials of pain treatments.” Investigators contemplating incorporating such methods into their trial should consider whether such testing may aid in explaining the hypothesized mechanism of action, optimize the trial in some fashion, or facilitate accelerated progression of intervention development.

### 3.2. Selected clinical conditions

Proof-of-concept trials designed to assess preliminary signal of treatment efficacy typically enroll patients with specific, well-defined chronic pain conditions that may serve as “model” pain states, and the results can later be extended more broadly to other related types of chronic pain. Appropriate selected chronic pain conditions should have well-established diagnostic criteria to enhance study uniformity and reproducibility, as well as enough prevalence to allow for adequate enrollment. The most common neuropathic pain conditions studied are painful diabetic peripheral neuropathy (DPN) and postherpetic neuralgia,^136^ but other peripheral neuropathic pain states such as HIV-associated neuropathy, post-traumatic neuralgia, lumbar radiculopathy, and chemotherapy-induced peripheral neuropathy have also been studied.^45,57^ Selected conditions of central neuropathic pain include poststroke pain and pain associated with spinal cord injuries. Rheumatoid arthritis is one of the most commonly used selected clinical conditions of inflammatory pain,^171^ whereas osteoarthritis has features of mechanical, inflammatory, and even, according to some researchers, neuropathic pain.^112^ Fibromyalgia is a classic example of a disorder of central sensitization, in which pain occurs even in the absence of tissue abnormalities.^152,157^

Unlike preclinical models, clinical conditions of pain demonstrate significant patient heterogeneity, both among and within specific pain conditions. Clinical pain in certain individuals may result from multiple mechanisms, making it more difficult to interpret and apply experimental findings. In addition, placebo group response in pain studies is typically quite high. In a review of neuropathic pain trials, studies with a high placebo response were less likely to demonstrate a positive treatment effect.^91^ There is some evidence that placebo group response may be affected by the pain condition studied; for example, placebo group improvement has been found to be significantly higher in DPN compared with postherpetic neuralgia.^48,136^ Thus, it is important to bear in mind the particular characteristics, strengths, and limitations of each potential model during the design and interpretation of POC trials. Models of chronic pain provide an important framework for understanding mechanisms of chronic pain and characterizing pain treatments, and they are a critical aspect of POC study design.

## 4. Research designs

Successful POC trials require substantial consideration in selecting the most appropriate research design for the particular research question, intervention, and outcome(s) of interest. The Initiative on Methods, Measurement, and Pain Assessment in Clinical Trials (IMMPACT) has published several recommendations and systematic reviews in recent years that have advanced the field and guided trials to be more cohesive. The group recently published POC consensus recommendations and an overview of research designs; the reader is directed to Gewandter et al.^63^ for a more thorough review of each method and detailed strengths and weaknesses of each approach, also briefly overviewed in Table 3. Design options for POC clinical trials are similar to those of larger clinical trials and frequently inform dosing, safety profile, preliminary signal of efficacy, selection of patient population, the specific assessments most meaningful to the population, as well as initial estimate of feasibility, all useful in planning for later phase studies. The most common research design elements used in POC trials are summarized here (Table 3).

**Table 3:**
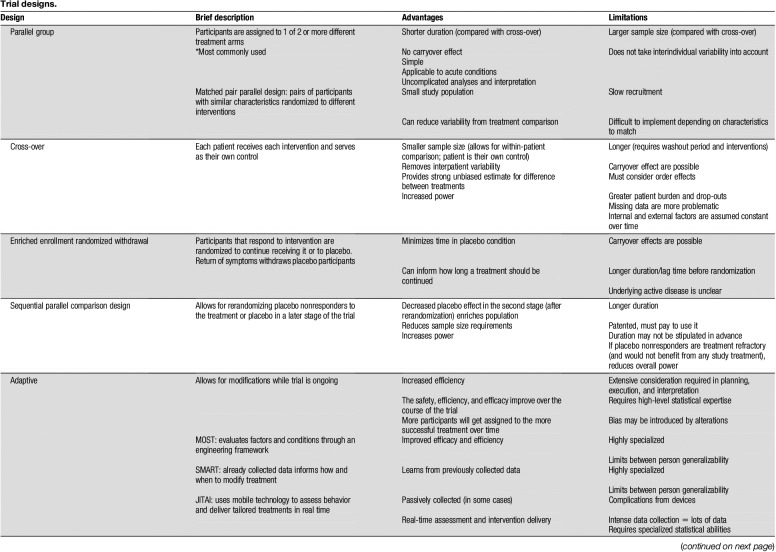

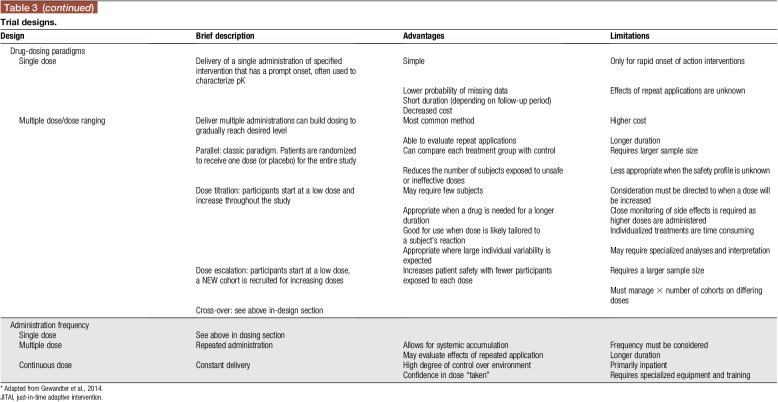
Trial designs.

### 4.1. Parallel group

Parallel group designs assign participants to one of the multiple treatment groups (eg, active treatment, placebo, and active comparator), and they remain in the assigned group throughout the duration of the trial. This design is particularly useful when drug effects are of lengthy or unknown duration; however, it requires larger sample sizes than cross-over trials (described below). To enhance the comparability of the placebo and intervention treatment groups, stratified randomization with blocking (or a similar technique) is recommended when possible. Blocking enhances balance in terms of the number of subjects allocated to each group. The number of stratification variables should be limited, and they should be variables that are strongly associated with outcome.

### 4.2. Cross-over

A cross-over design is a repeated-measurements (within-subject) design, in which each participant receives more than one different intervention during a specified period (ie, the patients “cross-over” from one treatment to another at a specified interval during the course of the trial). This design typically requires a “wash-out” period of sufficient length between ending one intervention and initiating another. Each participant therefore serves as his or her own control, providing greater statistical power for analyses with fewer participants than parallel group designs. Cross-over designs may yield a lower placebo response when compared with parallel group trials, hypothesized to result from participants receiving both the active and placebo treatments.^64,91^ However, given their temporal nature, cross-over designs are not suitable for treatments that have long-term effects or outcomes that cannot be measured relatively quickly, and particularly when an appropriate wash-out period is unknown or not feasible.

### 4.3. Enriched cohort

Enrichment designs encompass a number of strategies used to increase the likelihood that a drug effect will be detected if it exists. They focus on selecting appropriate participants (1) to decrease heterogeneity, such as selecting patients with specific characteristics to increase power, (2) to display the endpoints of interest (prognostic enrichment), and/or (3) that may be most likely to respond to the intervention (predictive enrichment).^55^

#### 4.3.1. Enriched enrollment randomized withdrawal

An enriched enrollment randomized withdrawal,^92,118^ a type of predictive enrichment strategy, randomizes participants to continued intervention or placebo based on their response. As such, this design excludes participants who are identified as potential nonresponders, have a specified magnitude of placebo response, cannot tolerate the experimental treatment, are noncompliant with treatment, etc. during an initial phase of the trial. Thus, they generally involve only patients who seem to have responded by some prespecified time point. The enriched enrollment randomized withdrawal trial design has been specifically suggested for early phase drug testing in humans.^83^ Such enrichment designs may improve assay sensitivity^83^ and potentially improve the likelihood of detecting patient subgroups that could benefit most from specified treatments, given the attention paid to responders. However, they may not necessarily improve the efficacy or efficiency of a trial (as they may require a longer overall study period to identify responders),^181^ may underestimate adverse effects,^59^ and could limit generalizability (although limited generalizability is not typically a concern for POC trials).^63^

### 4.4. Adaptive designs

Adaptive clinical trial designs allow for ongoing modifications to the trial based on observations and data generated at prespecified periods over the extant study period. This flexibility affords opportunities to alter procedural aspects of the study, as well as the statistical plan, after the trial has been initiated but before its completion.^63^ Such modern designs improve the efficiency of trials and potentially enhance understanding of treatment effects by allowing incoming data to guide refinement of the trial. For example, inappropriate dose selection is frequently a concern in drug development.^117^ An adaptive dose-finding POC trial might include several doses to evaluate dose–response and allocate additional patients to the doses showing greatest initial promise, thereby reducing the number of participants assigned to receive a less ideal dose. Similarly, group-sequential designs incorporate a framework for analyses, as data are collected; analyses are conducted, as data are collected; and continued trial enrollment is based on predefined stopping rules.^95^ Sequential parallel comparison designs allow for rerandomization of placebo nonresponders to treatment or placebo in a later stage of the trial.^3^ For example, placebo nonresponders are rerandomized to a second stage where they either receive placebo or active treatment, creating an additional subgroup for analysis and interpretation of true treatment effects. This design specifically seeks to address high placebo response rates, which is particularly relevant for chronic pain studies,^63^ and allow for additional data (specifically from placebo nonresponders), increasing the efficiency of the trial.

Despite their potential to improve trial efficiency, adaptive designs have not been widely reported in the extant literature.^81^ However, these study designs are gaining attention, and recent nonpharmacological work suggests an important place for them. For example, examining the points at which exposure to a specified intervention should occur or the order in which to introduce various aspects of one or more interventions represent an important and neglected area for nonpharmacological trials, which may be particularly suited to a POC trial. Multiphase optimization strategy (MOST) aims to improve the efficacy and efficiency of behavioral interventions through evaluating discrete factors and combinations of experimental conditions through an engineering framework.^180^ Sequential, multiple assignment, randomized trial (SMART) designs exploit collected data to inform decision making regarding how and when to modify a patient's treatment^102^ and may be included in a MOST design. It specifically seeks to optimize time-varying components of an intervention design, for example deciding the best sequence to deliver a series of intervention components. Limitations include being highly specialized and personal, so between-person generalizability is limited (not necessarily a problem for POC trials). Just-in-time adaptive interventions, perhaps the most cutting-edge clinical trial design, use smart phones, mobile computers, sensors, and software analytics to automatically detect an individual's behavior and deliver tailored treatment in real time.^164^ Although these innovative paradigms are gaining traction in mental health and substance abuse research, they have not yet been applied widely to the field of pain. A handful of pain studies that have applied such methods have focused on adapting stimulation parameters and position of sensors in neuromodulation studies.^122,145^ Expanding the use of adaptive designs and identifying decision rules that can guide the individualized sequence of intervention implementation could improve outcomes and advance personalized pain medicine.

Although novel and potentially valuable, adaptive designs do include multiple limitations and practical hurdles.^29,62,137^ The temporal framework, or how variables of interest interact and are ordered over time and across environments, can be challenging to discern, although strategies such as ecological momentary assessment provide opportunities to evaluate timescale.^121^ Ecological momentary assessment involves making repeated observations in real time, sometimes across a variety of contexts, eg, maintaining written or electronic pain diaries over a specified period. Related limitations include the logistical hassles of monitoring devices or techniques, procedural complications, and the overwhelming amount of data collected in such a study, requiring specialized analytic approaches. Another limitation specific to just-in-time adaptive interventions and ecological momentary assessment could be the perceived invasiveness of monitoring and the obstacle of collecting truthful, accurate information. This may be especially challenging in pain medication monitoring, given the current opioid crisis and potential participant concern regarding stricter oversight, and regulation. For additional information on innovative psychosocial clinical trials for pain, see “Unique aspects of clinical trials of psychosocial and integrative chronic pain treatments” by Kerns, Edmonds, Turk, and Williams.

### 4.5. Drug-dosing paradigms

#### 4.5.1. Single dose

Single-dose trials involve delivery of a single administration of the specified intervention, often randomized with placebo, and monitoring of analgesic effects. Single-dose administration is a feature that can be incorporated into a variety of POC trial designs, such as parallel-group, cross-over studies, or cohort studies, discussed above, or even as a smaller study within in the context of larger, repeated-dose trials. Such single-dose studies are frequently conducted as an initial step to evaluate the safety and preliminary efficacy for an acute pain medication. This design is particularly useful in determining effective dose ranges (as in single or multiple ascending dose studies), pharmacokinetics and pharmacodynamics, time to onset of effect, magnitude and duration of analgesic effects, and safety concerns. Single-dose trials have been used to evaluate short-term follow-up periods, as in the case of pretreatment before surgical interventions,^41^ as well as longer term follow-up, when drug effects are believed to last for prolonged periods. For example, high-concentration capsaicin can produce long-lasting pain relief.^20,123^ Single-dose studies are particularly recommended as an efficient screening method for future clinical trials when the treatment is expected to produce a rapid onset; however, they are less appropriate for addressing preliminary signal of treatment efficacy or adverse events when prolonged treatment is required.^63^ Single-dose methods of medication administrations, however, are also conducted within the context of other trial designs (parallel, cross-over, etc.; see below). Single ascending dose studies generally monitor participants and administer escalating doses until a predefined level, maximum exposure is reached, or intolerable side effects are observed.

Single-session treatments, similar to the concept of a single-dose trial, but outside of the pharmaceutical realm, are emerging. One such study found that healthy participants who underwent a brief, single cognitive-behavioral intervention evidenced reduced areas of secondary hyperalgesia to thermal stimuli compared with a control group.^142^ In a mixed etiology chronic pain study, patients were found to benefit from a single, ∼2-hour session of cognitive-behavioral therapy for pain catastrophizing, a negative mental set characterized by rumination, helplessness, and magnification of pain sensations.^34^ For additional information on psychosocial clinical trials for pain, see “Unique aspects of clinical trials of psychosocial and integrative chronic pain treatments” by Kerns, Edmonds, Turk, and Williams.

#### 4.5.2. Multiple dose/dose ranging

Dose-ranging studies involve administering different doses of an agent and analyzing each to evaluate the most effective dose with the fewest side effects. These include parallel dose comparison studies, where several potential doses are selected and subjects are randomized to receive one of the doses or placebo for the entire study; dose-titration studies, where a low dose is titrated up incrementally to the maximum tolerable dose, a predefined level, or to the onset of side effects; dose escalation, where a group is administered a starting dose and (when appropriate) a new cohort is recruited and administered a higher dose; and cross-over, where patients are administered 2 or more substances with a washout period, as described above. At predefined points or at the end of each study type, a comparison can be conducted between each treatment group and the control group to examine safety and efficacy. Each method has pros and cons (Table 3).^60,161,162^

#### 4.5.3. Administration frequency

Several drug administration regimens are available; single-dose administrations, as described above, multiple and continuous administration are the most frequently used. Repeated administration is the most common medication delivery regimen. In this approach to the maintenance of drug therapy, doses are taken at specific intervals; often desired accumulation occurs when the drug is administered before the previous dose is completely eliminated. The amount of drug within the system progressively rises. Dosing level and frequency are chosen (likely based on single-dose safety studies) to achieve therapeutic systemic drug levels and maintain a steady state, providing an opportunity to allow for monitoring of safety parameters. In multiple ascending dose studies patients receive low doses of the drug, which are subsequently escalated to a predetermined level. A “safety margin” may be determined from such dosing schedules when administered around a therapeutic window: continuous dosing, often continuous infusion, and delivers medication constantly for hours or days. It is most often conducted in cases of postoperative pain, severe cancer pain, or during vaso-occlusive crisis in patients with sickle cell disease or labor and delivery. These dosing regimens are infrequently conduced in POC trials.

## 5. Maximizing assay sensitivity in proof-of-concept trials

Providing first evidence of efficacy of a new treatment in a POC trial is facilitated by clinical trial factors that maximize trial assay sensitivity–defined as “the ability of an RCT to distinguish an effective treatment from a less effective or ineffective treatment.”^47^ Such factors may include (1) evaluating the maximally tolerated dose/intensity of the treatment; (2) using methods that minimize variability in outcome measurement; (3) studying a specific population (eg, postherpetic neuralgia vs a more heterogeneous group of neuropathic conditions); and, possibly, (4) adopting trial features that minimize nonspecific improvements often referred to as “placebo effects” but not necessarily limited to placebo-treated individuals. In the setting of POC trials, a “negative” trial would be considered a trial with an outcome that generates a “no-go” decision (ie, no evidence of analgesic efficacy–no reason to proceed to phase 3), and a “positive” trial would be considered a trial with an outcome that generates a “go” decision (ie, promising evidence of analgesic efficacy–supports proceeding to phase 3). Thus, a key objective of POC trials is to minimize the risk of a “false-negative” trial outcome, or not detecting benefits of efficacious treatments, while also considering the potential tradeoff of having a “false-positive” trial, or finding a benefit, ie, purely an artifact.^47,63^ Several strategies currently being investigated may help improve assay sensitivity in POC trials and other types of analgesic trials; these include (1) focused training of trial participants to more reliably rate their pain^154^; (2) limiting the number of clinical trial sites in multicenter trial with the expectation of reducing the magnitude of placebo response^47^; (3) excluding prospective trial participants with highly variable baseline pain levels^54^; and (4) restricting the use of concomitant analgesic treatments during clinical trials.^47^

## 6. Mechanism-based approach to analgesic trials

Confirming the specific target and mechanism of action for an investigational drug, based on preclinical animal data, is often the driving force behind POC trials. Disease-specific preclinical models that hope to reproduce pathophysiological conditions studied in humans have been developed, albeit with variable translational potency.^131^ However, personalized, mechanism-based treatment, while suggested nearly 30 years ago,^43,114,179^ has been slower to take shape. There has been an increasing recognition in recent years that substantial variability exists between patients, even with the same diagnoses, advancing the call for personalized pain medicine. Predicting the response to pain treatment has become an area of intense interest. This goal would incorporate genetic, demographic, and clinical phenotype information to deliver a specified intervention to those for which it might be most beneficial. Such identification could be used to group patients according to pain-related sensory profiles to enhance pain care. Recent work has outlined a number of recommendations for such profiling.^6,50,155^ Characterizing psychosocial factors, baseline pain report, within-patient variability in pain perception, underlying pain mechanism, behavioral measures such as sleep and fatigue, response to sensory testing/pain modulation profile, responses to pharmacological challenges, and genetic profile are all targets for population subgrouping. Predictive algorithms for identifying which—or which combination—of these factors might predict intervention efficacy is an exciting study frontier and well-suited for POC trials, given their exploratory nature.

Indeed, increasing attention has focused on predictive phenotyping before some specified treatment, often analgesic trials^49,51,75,125,185^ or surgical intervention.^77,139,176^ Presumably, such profiling could be of great clinical importance to identify target populations for whom the intervention of choice may have the greatest benefit, to recognize likely nonresponders and allocate supplemental resources to them or, in the case of modifiable risk factors, to develop alternate interventions to target the specified characteristics, potentially improving the likelihood of benefit in refractory groups.

### 6.1. Subgrouping patients

Historically, 50% of randomized clinical trials report at least one subgroup analysis.^134^ Guidelines have been proposed for evaluating and interpreting the results of subgroup analyses,^46^ which include evaluating the clinical importance of the difference, whether the hypotheses were stated a priori or were exploratory, whether the subgroups were limited in number, and if repeated, whether there is general consistency across studies.^126^ Typically, subgrouping is exploratory and should be interpreted with caution; however, unplanned subgroup analyses can be valuable to inform hypothesis generation for future study. Not surprisingly, larger, prospective studies are required to power subgroup analyses appropriately. Recent work has reviewed the challenges of postrandomization subgrouping.^40^ Although subgrouping at the POC stage should be conducted and interpreted with caution, the study population within a POC trial could be prospectively enriched to include those with the greatest likelihood benefiting.^172^

Predefining the mechanistic classification of patients to categorize likely responders is a developing area of considerable excitement. Although this manner of deep phenotyping, comprehensively assessing factors of interest, has spurred a number of studies exploring postoperative pain outcomes^12,28^ and at least one large population-based study to identify characteristics that contribute to the onset and persistence of pain,^111^ POC and other clinical trials have been slower to use these concepts. Recent IMMPACT meetings have focused on improving assay sensitivity,^47^ patient phenotyping in clinical trials of chronic pain,^50^ and on specific viable biomarkers, including sensory testing, skin punch biopsy, and brain imaging, suggesting a number of promising tools for incorporation into clinical trial design.^155^ Here, we briefly summarize some of the research to date that focuses on baseline characterization of pain mechanisms and their impact on treatment response.

### 6.2. Genetic profile

The extent to which genetic factors impact patient response to treatment is an area of substantial interest. Identifying the genetic factors that contribute to variability in opioid efficacy, metabolism, and adverse effects will advance personalized pain management, with the future objective of point-of-care genotyping to assist clinicians in personalizing drug-dosing regimen to each individual. Rodent models have produced hundreds of candidate pain genes (http://www.jbldesign.com/jmogil/enter.html), and genetic association studies have evaluated how single-nucleotide polymorphisms are associated with clinical pain and pain sensitivity.^159,173^ Evaluation of genetic factors and their potential in informing analgesic choice or dosing strategy has been reviewed comprehensively,^14,27,36,58,99,119,148,153,166,173^ and new studies are exploring genetic subgroups in treatment efficacy and safety.^135^ Generally, genetic association studies examining drug response have not yielded conclusive guidance on treatment. Epigenetic studies may aid in addressing some of the dynamic gene-by-environment interactions that likely play a role in pain generation and chronification.^4,39^ Clinical trials designed to include genetic analysis could be extremely useful in patient subgrouping to improve drug efficacy, reduce side effects, and ultimately optimize pain management. Given the smaller sample size of POC trials and the logistics and cost of collecting and processing DNA, such genetic subgrouping can be exploited in POC trials by only including participants with the variants of choice.

Perhaps the most progress has been made in understanding the influence of the drug metabolism pathways, particularly the cytochrome p450 system, on both analgesic efficacy and adverse effects. A small “pharmacokinomic” randomized, cross-over, double-blind, placebo-controlled trial in healthy men found that an individual's CYP2D6 genotype (categorizing them into metabolizer phenotypes) impacted the relationship between oxycodone dose, expected plasma levels, and the therapeutic range, offering dosing guidelines based on genotype.^106^ Although this assessment had notable limitations,^98,141^ it attempts to merge genomic and pharmacokinetics to advance personalized patient care. Similar work has been performed in assessing codeine and methadone.^61,96^ Another ongoing study in chronic low back pain is seeking to link genetic polymorphisms of cytochrome p450 enzymes and other relevant pain processing molecules, as well as sensory testing responses, to tricyclic antidepressant, opioid agonist, and GABA_A_-agonist treatment effects.^148^ Such studies are time- and resource-intensive but necessary as a step toward individualized pain care. Nevertheless, because of the large sample sizes required to elucidate DNA's contribution to drug response, genetic profiling has limited utility in POC trials until more conclusive work reveals the specific polymorphisms or clusters of single-nucleotide polymorphisms, and potentially interaction with other characteristics, that could modulate treatment effects.

### 6.3. Sensory phenotypes

The association between various QST measures and clinical pain has been well-documented, both in connection to acute and chronic pain perception, sensitivity in forecasting clinical deterioration, as well as prediction of postoperative pain outcomes in a variety of surgical procedures.^32,71,128,175,182–184,186^ Emerging evidence suggests that nociceptive characteristics may serve as predictors of response to a number of nonpharmacological interventions including multidisciplinary pain treatment,^50^ spinal pain outcomes,^31^ and spinal cord stimulation outcomes.^21^ Although few clinical trials have taken advantage of this approach,^50^ academic endeavors suggest promising opportunities.^16^ Several reviews have recently summarized the utility of QST in quantifying sensory function and its potential value in selecting patients that might be most appropriate for a certain intervention.^32,50,75,94,155,160,169,184^ In 2013, Grosen et al.^75^ comprehensively reviewed the extant QST literature specific to predicting response to analgesic treatment.

In brief, baseline QST responses have been associated with the efficacy of lidocaine, lamotrigine, pregabalin, oxycodone, oxcarbazepine, and placebo analgesia.^50^ In a multicenter observational cohort study, Grosen et al.^76^ found that opioid response was predicted by cold pain intensity, pain catastrophizing, and beta EEG activity induced by laboratory cold pain in a small sample of mixed-type chronic pain patients. Pretreatment pain inhibition, often measured through conditioned pain modulation (counterirritation believed to reflect descending pain control^156^), has been associated with postoperative pain outcomes,^13,183^ the benefits of exercise,^103^ morphine consumption after chest wall surgery,^77^ duloxetine benefit in painful diabetic neuropathy patients,^185^ and NSAID efficacy.^49^

Prespecified QST hypotheses have recently emerged in a handful of study designs. For example, some used QST to identify an “irritable nociceptor” subgroup, or sensory hyperexcitability, and evaluated whether the specified intervention had differential efficacy based on this group membership.^7,22,37,38^ This concept is nicely illustrated by Demant et al.,^38^ who observed greater analgesic efficacy of oxcarbazepine for neuropathic pain in an “irritable nociceptor” sensory phenotype subgroup, determined through comprehensive QST battery to identify those with sensory gain, vs no efficacy in the “nonirritable nociceptor” subgroup. Such subclassification of patients at baseline has produced excitement but has been met with mixed results in other clinical analgesic trials.^22,35,37,82,93,110^ As recently discussed by Dworkin and Edwards,^44^ these studies contain important methodological differences, including assessment of a single active treatment, comparison between active and placebo interventions, and retrospective analyses, so the exact role of QST in guiding study design and treatment decisions has yet to be firmly established. Nevertheless, these findings show promise in eventually elucidating QST-identified, shared underlying pain mechanisms that would impact treatment response and/or selection of advantageous subgroups, but the vast heterogeneity of conditions, outcomes, and QST methods have proved challenging in moving routine QST characterization into trials.^169^

### 6.4. Psychobehavioral profile

Psychosocial and behavioral characteristics and how they may impact treatment outcomes have been reviewed recently with recommendations for including specific measures in clinical trials.^50^ A few more recent studies continue to advance such assessment. In an evaluation of postoperative opioid consumption after hysterectomy, Janda et al.^88^ found that, after controlling for other potential predictors, a 1-point increase in fibromyalgia survey scores (based on the 2011 criteria) were associated with an increase of 7-mg oral morphine equivalents. Interestingly, those scoring in the top third of the survey required nearly 30% more opioids than those scoring in the bottom third. These findings replicate previous work, finding that fibromyalgia survey score predicted enhanced opioid requirements after total knee and hip arthroplasty.^19^ In an elegant series of studies, Booth et al. identified 3 questions, answered before cesarean delivery, that predicted postcesarean evoked pain.^129^ These questions included assessment of anxiety and anticipated pain level and analgesic use. In a subsequent study, the investigators randomized patients endorsing elevated risk for postoperative pain, based on responses to their preoperative survey (“enriched population”), into a clinical trial where they received usual care or additional analgesic treatment (higher dose of spinal morphine combined with systemic acetaminophen and IV PCA).^11^ They found that this adjunct treatment significantly reduced acute pain scores at 24 hours, as well as pain on movement and average pain report.

### 6.5. Opioid receptor function/pharmacological challenge

Through sophisticated naloxone blockade studies, Bruehl et al. have found that endogenous opioid inhibition influences morphine efficacy. Specifically, in a randomized, counterbalanced, cross-over (3 single dose: morphine, naloxone, and placebo) study, they found that morphine efficacy is moderated by endogenous opioid function (evaluated through QST) in healthy participants and low-back pain patients.^17^ They confirmed this effect in a larger sample of chronic low-back pain patients, specifically finding that those with greater natural endogenous opioid inhibition experience less acute relief of back pain with morphine.^18^ A number of studies have evaluated how early response to a medication predicts long-term response, as well as infusion screening of IV lidocaine and ketamine in forecasting analgesic benefit (see Ref. 50 for review).

Proof-of-concept trials, given their exploratory nature, are uniquely suited to prespecify logical, mechanism-based treatment modifiers in the effort to advance personalized pain treatment, which can be assessed more thoroughly in larger, later-stage trials. Identifying biomarkers, potentially based on pathophysiological/psychobehavioral mechanisms, could inform study populations, appropriate subgroups, or new indications that will aid in customizing interventions and guide treatment choices. The logical next step would be the inclusion of systematic phenotyping routinely in trials to advance or refute such a symptom-/mechanistic-based treatment approach.

### 6.6. Sex and gender

Over the past several decades, researchers have developed a deeper understanding of sex- and gender-related influences on clinical pain. A number of studies have provided evidence that pain processing may be different between men and women in response to both experimentally induced and clinical pain conditions.^8,74,115,127,140^ Various research and professional organizations have advocated for more research into the effects of sex and gender on pain, as well as for the inclusion of women in both preclinical and clinical research studies.^56,120^ Consequently, POC analgesic trials should consider study in both sexes.

Subgrouping patients by sex can shape numerous aspects in the design and interpretation of POC trials. Possible sex differences in response to experimental pain models may either limit the target patient population or broaden the overall generalizability of a study's findings, thus guiding future studies. For example, one study of experimental endotoxemia as a model for inflammatory pain suggested that pain perception and modulation are more sensitive to immune activation in women than in men,^90^ whereas another group found no sex differences in endotoxin-induced pain sensitization.^174^ Researchers considering the use of such pain models must therefore carefully consider how sex may influence interpretation of findings. Another example of the potential value of studying experimental responses to pain in both sexes is the study of the placebo effect, which has important implications for clinical trial design based on expected response to placebo. Several studies have observed small, but significant differences in placebo effects and pain processing between men and women.^80,163^ Finally, an increasing number of studies are evaluating the effect of patient sex on clinical pain outcomes in response to a variety of analgesics, from opioids^89^ to cannabis.^30^ Such studies can provide greater insights about which patients are most likely to benefit from which therapies, adding an important element to the development of personalized pain medicine.

## 7. Statistical issues

The nature of POC studies, with their small sample sizes and fewer endpoints, presents statistical challenges. Smaller sample sizes allow for easier recruitment, lower cost, and more efficient completion of a clinical trial, at the expense of diminished statistical power and potential inability to detect clinically significant effects.^146^ Therefore, POC studies typically need to deviate from the standard α (significance level or type I error probability) of 0.05 and β (type II error probability) of 0.1 (ie, 90% power) to remain cost-effective^25^ and may require more advanced statistical analysis techniques.^25^ In the IMMPACT recommendations on research designs for proof-of-concept chronic pain trials, an instructive example is given: consider 2 different chronic pain conditions, painful DPN vs pain HIV neuropathy.^63^ In a study of painful DPN, a higher type II error probability (false-negative) may be more acceptable because other efficacious treatments are available, whereas HIV neuropathy has very few efficacious treatments, and accepting a higher type I error probability (false-positive) would decrease the risk of missing a potentially beneficial therapy.

Because small sample sizes give individual subjects significant influence on study outcomes, appropriate participant selection is crucial to the success of a POC study. For POC trials evaluating preliminary treatment efficacy, appropriate inclusion and exclusion criteria must be formulated based on the POC to be studied, and these criteria must be rigorously applied to create appropriate homogeneity, thus maximizing statistical power and efficiency. By contrast, POC trials designed to identify target treatment populations may necessarily have a heterogeneous patient population, yet a small sample size would yield low power to detect a treatment effect in each subgroup. In such cases, an N-of-1 or cross-over study design may be more appropriate than the traditional parallel-group trial, although these may not always be feasible depending on the pain condition or treatment being studied.^63^

Another important distinction between POC trials and confirmatory trials is the use of “early efficacy endpoints,” as opposed to clinical endpoints.^26,116^ For example, a POC pain study may assess a decrease in area of mechanical hyperalgesia as measured by QST for its primary endpoint rather than a decrease in pain score. The early endpoints used in POC trials theoretically have larger treatment effect sizes and can be assessed in shorter periods, allowing for smaller sample sizes to achieve adequate statistical power and faster evaluation of preliminary efficacy. However, appropriate early efficacy endpoints may not always exist, and even when they do, they may not correlate with meaningful clinical outcomes. Researchers should therefore carefully consider whether an early efficacy endpoint may be appropriate for their potential study, and furthermore, whether the increased potential for identifying analgesic efficacy will translate to significant clinical results in later trials.

Adaptive designs are another approach used in POC trials to reach meaningful conclusions in a shorter period than traditional clinical trials. As discussed previously, adaptive designs, such as adaptive dose-finding designs, adaptive allocation designs, group-sequential designs, and sample-size re-estimation designs allow for changes in study protocol and statistical analysis as new data are acquired.^63,161^ These changes may include adjusting randomization ratios or treatment allocation, modifying protocols, or changing sample size; such changes may increase the potential for bias or reduce the overall statistical power of the study. However, the ability to perform interim analyses and respond accordingly may be critical to the overall success of the trial and may even help determine whether the trial should be continued. Therefore, adaptive designs require extensive planning and careful consideration of the many logistical and procedural challenges that may impede modifications to an ongoing study.^80^ The precise nature and timing of all protocol changes and interim data analyses must be planned and described in the protocol before the initiation of the study to minimize potential errors in trial results, allow for clear interpretation of data, and provide valid conclusions.^104^

As with any study with a small sample size, conclusions drawn from a POC study may be difficult to generalize to a larger patient population. In addition, small studies are less likely to pick up rare but serious adverse effects that may only later be detected in much larger clinical trials. However, taking POC studies for what they are—limited, small-scale studies addressing a focused research area—provides a strong basis for future research and new opportunities.

## 8. Summary/future directions

Traditionally, POC clinical trials are studies where a drug (device or method; such as high-frequency spinal cord stimulation^1^) is examined for the first time for its biologic activity, efficacy, and safety in patients. For new molecular entities, POC trials are an essential component of the “exploratory development” phase that helps make the critical go/no-go decision—whether to embark on a larger, definitive clinical trial or to avoid wasting resources in a study that is likely to fail. The meaningful interpretation of POC trials of new drugs for pain requires evidence that the drug reaches the target (receptor occupancy), the drug affects the target (target engagement), and the drug affects pain signaling mechanisms in a dose-dependent manner.

Proof-of-concept trials have also been used as a research tool in the development and validation of new “pain models” and pain-related outcome measures, identification of physiological and pathological pain mechanisms, evaluation of biomarkers to predict chronic pain treatment outcomes, assessing the preliminary efficacy and safety of treatment strategies such as combination therapy, and others. Several preclinical and clinical models of chronic pain have been used to help determine the appropriate target patient population for POC trials and the presence of an “analgesic signal.” Both human experimental pain and clinical models have their strengths and limitations, and the appropriate model should be selected based on the understanding of the mechanism of action of the drug being tested.

The design of a successful POC trial requires careful consideration of the research objective, patient population, the particular intervention, and outcome(s) of interest. Proof-of-concept studies have used a variety of study designs in an attempt to enhance assay sensitivity and minimize the risk of a “false-negative” trial outcome. Although no one design may be uniformly applicable, enriched enrollment and adaptive designs may improve assay sensitivity and the efficiency of trials.

A challenge for future studies is adapting POC trials to address the emerging initiatives toward personalized/precision medicine. Personalization of pain management would require better insights on pain mechanisms in a given individual (phenotype), genetic factors (genotype), environmental, and behavioral factors influencing the pain experience. Although precision medicine is a worthwhile future goal, it adds a complexity to the design of appropriate studies that may require innovative large-scale research approaches.

## Disclosures

C.M. Campbell has received grants from NIH. I. Gilron has received support from Biogen, Adynxx, TARIS Biomedical, AstraZeneca, Pfizer, and Johnson and Johnson and has received grants from the Canadian Institutes of Health Research, Physicians' Services Incorporated Foundation, and Queen's University. S. Raja has received grants from NIH and Medtronic, Inc., and funding from Allergan and Aptinyx outside the submitted work. The remaining author has no conflicts of interest to disclose.

This work was supported in part by NIH grants DA-042751 (C.M.C.) and NS-26363 (S.R.).

## References

[1] Al-Kaisy A Palmisani S Smith TE Pang D Lam K Burgoyne W Houghton R Hudson E Lucas J 10 kHz high-frequency spinal cord stimulation for chronic axial low back pain in patients with no history of spinal surgery: a preliminary, prospective, open label and proof-of-concept study. Neuromodulation 2017;20:63–70. 28025843 10.1111/ner.12563

[2] Arendt-Nielsen L Chen AC Lasers and other thermal stimulators for activation of skin nociceptors in humans. Neurophysiol Clin 2003;33:259–68. 14678840 10.1016/j.neucli.2003.10.005

[3] Baer L Ivanova A When should the sequential parallel comparison design be used in clinical trials? Clin Invest 2013;3:823–833.

[4] Bai G Ren K Dubner R Epigenetic regulation of persistent pain. Transl Res 2015;165:177–99. 24948399 10.1016/j.trsl.2014.05.012 PMC4247805

[5] Baron R Dickenson AH Neuropathic pain: precise sensory profiling improves treatment and calls for back-translation. PAIN 2014;155:2215–17. 25168667 10.1016/j.pain.2014.08.021

[6] Baron R Forster M Binder A Subgrouping of patients with neuropathic pain according to pain-related sensory abnormalities: a first step to a stratified treatment approach. Lancet Neurol 2012;11:999–1005. 23079556 10.1016/S1474-4422(12)70189-8

[7] Baron R Maier C Attal N Binder A Bouhassira D Cruccu G Finnerup NB Haanpaa M Hansson P Hullemann P Jensen TS Freynhagen R Kennedy JD Magerl W Mainka T Reimer M Rice AS Segerdahl M Serra J Sindrup S Sommer C Tolle T Vollert J Treede RD Peripheral neuropathic pain: a mechanism-related organizing principle based on sensory profiles. PAIN 2017;158:261–72. 27893485 10.1097/j.pain.0000000000000753 PMC5266425

[8] Bartley EJ King CD Sibille KT Cruz-Almeida Y Riley JL III Glover TL Goodin BR Sotolongo AS Herbert MS Bulls HW Staud R Fessler BJ Redden DT Bradley LA Fillingim RB Enhanced pain sensitivity among individuals with symptomatic knee osteoarthritis: potential sex differences in central sensitization. Arthritis Care Res (Hoboken) 2016;68:472–80. 26434740 10.1002/acr.22712 PMC4808376

[9] Bellamy N Buchanan WW Goldsmith CH Campbell J Stitt LW Validation study of WOMAC: a health status instrument for measuring clinically important patient relevant outcomes to antirheumatic drug therapy in patients with osteoarthritis of the hip or knee. J Rheumatol 1988;15:1833–40. 3068365

[10] Bickel A Dorfs S Schmelz M Forster C Uhl W Handwerker HO Effects of antihyperalgesic drugs on experimentally induced hyperalgesia in man. PAIN 1998;76:317–25. 9718250 10.1016/S0304-3959(98)00062-1

[11] Booth JL Harris LC Eisenach JC Pan PH A randomized controlled trial comparing two multimodal analgesic techniques in patients predicted to have severe pain after cesarean delivery. Anesth Analg 2016;122:1114–19. 25806400 10.1213/ANE.0000000000000695 PMC5223090

[12] Borges NC Pereira LV de Moura LA Silva TC Pedroso CF Predictors for moderate to severe acute postoperative pain after cesarean section. Pain Res Manag 2016;2016:5783817. 27956847 10.1155/2016/5783817 PMC5121467

[13] Bouwense SA Ahmed AU ten Broek RP Issa Y van Eijck CH Wilder-Smith OH van GH Altered central pain processing after pancreatic surgery for chronic pancreatitis. Br J Surg 2013;100:1797–804. 24227367 10.1002/bjs.9322

[14] Branford R Droney J Ross JR Opioid genetics: the key to personalized pain control? Clin Genet 2012;82:301–10. 22780883 10.1111/j.1399-0004.2012.01923.x

[15] Brennum J Dahl JB Moiniche S Arendt-Nielsen L Quantitative sensory examination of epidural anaesthesia and analgesia in man: effects of pre- and post-traumatic morphine on hyperalgesia. PAIN 1994;59:261–71. 7892024 10.1016/0304-3959(94)90079-5

[16] Bruehl S Personalized pain medicine: pipe dream or reality? Anesthesiology 2015;122:967–8. 25751235 10.1097/ALN.0000000000000638 PMC4439303

[17] Bruehl S Burns JW Gupta R Buvanendran A Chont M Kinner E Schuster E Passik S France CR Endogenous opioid function mediates the association between laboratory-evoked pain sensitivity and morphine analgesic responses. PAIN 2013;154:1856–64. 23748117 10.1016/j.pain.2013.06.002 PMC4069065

[18] Bruehl S Burns JW Gupta R Buvanendran A Chont M Schuster E France CR Endogenous opioid inhibition of chronic low-back pain influences degree of back pain relief after morphine administration. Reg Anesth Pain Med 2014;39:120–5. 24553304 10.1097/AAP.0000000000000058 PMC3933525

[19] Brummett CM Janda AM Schueller CM Tsodikov A Morris M Williams DA Clauw DJ Survey criteria for fibromyalgia independently predict increased postoperative opioid consumption after lower-extremity joint arthroplasty: a prospective, observational cohort study. Anesthesiology 2013;119:1434–43. 24343289 10.1097/ALN.0b013e3182a8eb1f PMC3867739

[20] Campbell CM Diamond R Schmidt WK Kelly M Allen R Houghton W Brady KL Campbell JN A randomized, double blind, placebo controlled trial of injected capsaicin for pain in Morton's neuroma. PAIN 2016;157:1297–304. 26963851 10.1097/j.pain.0000000000000544

[21] Campbell CM Jamison RN Edwards RR Psychological screening/phenotyping as predictors for spinal cord stimulation. Curr Pain Headache Rep 2013;17:307. 23247806 10.1007/s11916-012-0307-6 PMC3601592

[22] Campbell CM Kipnes MS Stouch BC Brady KL Kelly M Schmidt WK Petersen KL Rowbotham MC Campbell JN Randomized control trial of topical clonidine for treatment of painful diabetic neuropathy. PAIN 2012;153:1815–23. 22683276 10.1016/j.pain.2012.04.014 PMC3413770

[23] Cavallone LF Frey K Montana MC Joyal J Regina KJ Petersen KL Gereau RW Reproducibility of the heat/capsaicin skin sensitization model in healthy volunteers. J Pain Res 2013;6:771–84. 24232380 10.2147/JPR.S53437 PMC3827105

[24] Chaparro LE Wiffen PJ Moore RA Gilron I Combination pharmacotherapy for the treatment of neuropathic pain in adults. Cochrane Database Syst Rev 2012;CD008943. 22786518 10.1002/14651858.CD008943.pub2 PMC6481651

[25] Chen C Beckman RA Maximizing return on socioeconomic investment in phase II proof-of-concept trials. Clin Cancer Res 2014;20:1730–4. 24526732 10.1158/1078-0432.CCR-13-2312

[26] Chen C Sun L Li CL Evaluation of early efficacy endpoints for proof-of-concept trials. J Biopharm Stat 2013;23:413–24. 23437947 10.1080/10543406.2011.616969

[27] Chianta M Guevara M Pharmacogenetics and pain management: an opportunity to advance personalized patient care. MLO Med Lab Obs 2014;46:11. 24672845

[28] Chodor P Kruczynski J Predicting persistent unclear pain following primary total knee arthroplasty. Ortop Traumatol Rehabil 2016;18:527–36. 28155832 10.5604/15093492.1230507

[29] Coffey CS Levin B Clark C Timmerman C Wittes J Gilbert P Harris S Overview, hurdles, and future work in adaptive designs: perspectives from a National Institutes of Health-funded workshop. Clin Trials 2012;9:671–80. 23250942 10.1177/1740774512461859 PMC5570450

[30] Cooper ZD Haney M Sex-dependent effects of cannabis-induced analgesia. Drug Alcohol Depend 2016;167:112–20. 27522535 10.1016/j.drugalcdep.2016.08.001 PMC5037015

[31] Coronado RA Bialosky JE Robinson ME George SZ Pain sensitivity subgroups in individuals with spine pain: potential relevance to short-term clinical outcome. Phys Ther 2014;94:1111–22. 24764070 10.2522/ptj.20130372 PMC4118073

[32] Cruz-Almeida Y Fillingim RB Can quantitative sensory testing move us closer to mechanism-based pain management? Pain Med 2014;15:61–72. 24010588 10.1111/pme.12230 PMC3947088

[33] Dahl JB Brennum J Arendt-Nielsen L Jensen TS Kehlet H The effect of pre- versus postinjury infiltration with lidocaine on thermal and mechanical hyperalgesia after heat injury to the skin. PAIN 1993;53:43–51. 8316389 10.1016/0304-3959(93)90054-S

[34] Darnall BD Sturgeon JA Kao MC Hah JM Mackey SC From catastrophizing to recovery: a pilot study of a single-session treatment for pain catastrophizing. J Pain Res 2014;7:219–26. 24851056 10.2147/JPR.S62329 PMC4008292

[35] Davis KD Treede RD Raja SN Meyer RA Campbell JN Topical application of clonidine relieves hyperalgesia in patients with sympathetically maintained pain. PAIN 1991;47:309–17. 1664508 10.1016/0304-3959(91)90221-I

[36] DeFeo K Sykora K Eley S Vincent D How does pharmacogenetic testing alter the treatment course and patient response for chronic-pain patients in comparison with the current “trial-and-error” standard of care? J Am Assoc Nurse Pract 2014;26:530–6. 25132680 10.1002/2327-6924.12154

[37] Demant DT Lund K Finnerup NB Vollert J Maier C Segerdahl MS Jensen TS Sindrup SH Pain relief with lidocaine 5% patch in localized peripheral neuropathic pain in relation to pain phenotype: a randomised, double-blind, and placebo-controlled, phenotype panel study. PAIN 2015;156:2234–44. 26090758 10.1097/j.pain.0000000000000266

[38] Demant DT Lund K Vollert J Maier C Segerdahl M Finnerup NB Jensen TS Sindrup SH The effect of oxcarbazepine in peripheral neuropathic pain depends on pain phenotype: a randomised, double-blind, placebo-controlled phenotype-stratified study. PAIN 2014;155:2263–73. 25139589 10.1016/j.pain.2014.08.014

[39] Denk F McMahon SB Chronic pain: emerging evidence for the involvement of epigenetics. Neuron 2012;73:435–44. 22325197 10.1016/j.neuron.2012.01.012 PMC3996727

[40] Desai M Pieper KS Mahaffey K Challenges and solutions to pre- and post-randomization subgroup analyses. Curr Cardiol Rep 2014;16:531. 25135344 10.1007/s11886-014-0531-2 PMC4200313

[41] Dirks J Fredensborg BB Christensen D Fomsgaard JS Flyger H Dahl JB A randomized study of the effects of single-dose gabapentin versus placebo on postoperative pain and morphine consumption after mastectomy. Anesthesiology 2002;97:560–4. 12218520 10.1097/00000542-200209000-00007

[42] Drewes AM Schipper KP Dimcevski G Petersen P Andersen OK Gregersen H Arendt-Nielsen L Multimodal assessment of pain in the esophagus: a new experimental model. Am J Physiol Gastrointest Liver Physiol 2002;283:G95–103. 12065296 10.1152/ajpgi.00496.2001

[43] Dworkin RH Mechanism-based treatment of pain. PAIN 2012;153:2300. 10.1016/j.pain.2012.08.013 23006799

[44] Dworkin RH Edwards RR Phenotypes and treatment response: it's difficult to make predictions, especially about the future. PAIN 2017;158:187–9. 28092645 10.1097/j.pain.0000000000000771

[45] Dworkin RH O'Connor AB Audette J Baron R Gourlay GK Haanpaa ML Kent JL Krane EJ Lebel AA Levy RM Mackey SC Mayer J Miaskowski C Raja SN Rice AS Schmader KE Stacey B Stanos S Treede RD Turk DC Walco GA Wells CD Recommendations for the pharmacological management of neuropathic pain: an overview and literature update. Mayo Clin Proc 2010;85:S3–14. 10.4065/mcp.2009.0649 PMC2844007 20194146

[46] Dworkin RH Turk DC McDermott MP Peirce-Sandner S Burke LB Cowan P Farrar JT Hertz S Raja SN Rappaport BA Rauschkolb C Sampaio C Interpreting the clinical importance of group differences in chronic pain clinical trials: IMMPACT recommendations. PAIN 2009;146:238–44. 19836888 10.1016/j.pain.2009.08.019

[47] Dworkin RH Turk DC Peirce-Sandner S Burke LB Farrar JT Gilron I Jensen MP Katz NP Raja SN Rappaport BA Rowbotham MC Backonja MM Baron R Bellamy N Bhagwagar Z Costello A Cowan P Fang WC Hertz S Jay GW Junor R Kerns RD Kerwin R Kopecky EA Lissin D Malamut R Markman JD McDermott MP Munera C Porter L Rauschkolb C Rice AS Sampaio C Skljarevski V Sommerville K Stacey BR Steigerwald I Tobias J Trentacosti AM Wasan AD Wells GA Williams J Witter J Ziegler D Considerations for improving assay sensitivity in chronic pain clinical trials: IMMPACT recommendations. PAIN 2012;153:1148–58. 22494920 10.1016/j.pain.2012.03.003

[48] Dworkin RH Turk DC Peirce-Sandner S McDermott MP Farrar JT Hertz S Katz NP Raja SN Rappaport BA Placebo and treatment group responses in postherpetic neuralgia vs. painful diabetic peripheral neuropathy clinical trials in the REPORT database. PAIN 2010;150:12–16. 20202753 10.1016/j.pain.2010.02.002

[49] Edwards RR Dolman AJ Martel MO Finan PH Lazaridou A Cornelius M Wasan AD Variability in conditioned pain modulation predicts response to NSAID treatment in patients with knee osteoarthritis. BMC Musculoskelet Disord 2016;17:284. 27412526 10.1186/s12891-016-1124-6 PMC4944243

[50] Edwards RR Dworkin RH Turk DC Angst MS Dionne R Freeman R Hansson P Haroutounian S Arendt-Nielsen L Attal N Baron R Brell J Bujanover S Burke LB Carr D Chappell AS Cowan P Etropolski M Fillingim RB Gewandter JS Katz NP Kopecky EA Markman JD Nomikos G Porter L Rappaport BA Rice AS Scavone JM Scholz J Simon LS Smith SM Tobias J Tockarshewsky T Veasley C Versavel M Wasan AD Wen W Yarnitsky D Patient phenotyping in clinical trials of chronic pain treatments: IMMPACT recommendations. PAIN 2016;157:1851–71. 27152687 10.1097/j.pain.0000000000000602 PMC5965275

[51] Edwards RR Haythornthwaite J Tella P Max MB Raja SN Basal heat pain thresholds predict opioid analgesia in patients with post-herpetic neuralgia. Anesthesiology 2006;104:1243–8. 16732096 10.1097/00000542-200606000-00020

[52] Eisenberg E Midbari A Haddad M Pud D Predicting the analgesic effect to oxycodone by “static” and “dynamic” quantitative sensory testing in healthy subjects. PAIN 2010;151:104–9. 20621419 10.1016/j.pain.2010.06.025

[53] Enck P Benedetti F Schedlowski M New insights into the placebo and nocebo responses. Neuron 2008;59:195–206. 18667148 10.1016/j.neuron.2008.06.030

[54] Farrar JT Troxel AB Haynes K Gilron I Kerns RD Katz NP Rappaport BA Rowbotham MC Tierney AM Turk DC Dworkin RH Effect of variability in the 7-day baseline pain diary on the assay sensitivity of neuropathic pain randomized clinical trials: an ACTTION study. PAIN 2014;155:1622–31. 24831421 10.1016/j.pain.2014.05.009

[55] FDA. Enrichment strategies for clinical trials to support approval of human drug and biological products. Rockville, MD: The Federal Register, 2012 Available at: https://www.fda.gov/downloads/drugs/guidancecomplianceregulatoryinformation/guidances/ucm332181.pdf.

[56] Fillingim RB King CD Ribeiro-Dasilva MC Rahim-Williams B Riley JL III Sex, gender, and pain: a review of recent clinical and experimental findings. J Pain 2009;10:447–85. 19411059 10.1016/j.jpain.2008.12.001 PMC2677686

[57] Finnerup NB Sindrup SH Jensen TS The evidence for pharmacological treatment of neuropathic pain. PAIN 2010;150:573–81. 20705215 10.1016/j.pain.2010.06.019

[58] Fudin J Atkinson TJ Personalized oxycodone dosing: using pharmacogenetic testing and clinical pharmacokinetics to reduce toxicity risk and increase effectiveness. Pain Med 2014;15:723–5. 24636013 10.1111/pme.12417

[59] Furlan A Chaparro LE Irvin E Mailis-Gagnon A A comparison between enriched and nonenriched enrollment randomized withdrawal trials of opioids for chronic noncancer pain. Pain Res Manag 2011;16:337–51. 22059206 10.1155/2011/465281 PMC3206784

[60] Garrett-Mayer E The continual reassessment method for dose-finding studies: a tutorial. Clin Trials 2006;3:57–71. 16539090 10.1191/1740774506cn134oa

[61] Gasche Y Daali Y Fathi M Chiappe A Cottini S Dayer P Desmeules J Codeine intoxication associated with ultrarapid CYP2D6 metabolism. N Engl J Med 2004;351:2827–31. 15625333 10.1056/NEJMoa041888

[62] Gaydos B Anderson KM Berry D Burnham N Chuang-Stein C Dudinak J Fardipour P Gallo P Givens S Lewis R Maca J Pinheiro J Pritchett Y Krams M Good practices for adaptive clinical trials in pharmaceutical product development. Ther Innovation Regul Sci 2009;43:539–56.

[63] Gewandter JS Dworkin RH Turk DC McDermott MP Baron R Gastonguay MR Gilron I Katz NP Mehta C Raja SN Senn S Taylor C Cowan P Desjardins P Dimitrova R Dionne R Farrar JT Hewitt DJ Iyengar S Jay GW Kalso E Kerns RD Leff R Leong M Petersen KL Ravina BM Rauschkolb C Rice AS Rowbotham MC Sampaio C Sindrup SH Stauffer JW Steigerwald I Stewart J Tobias J Treede RD Wallace M White RE Research designs for proof-of-concept chronic pain clinical trials: IMMPACT recommendations. PAIN 2014;155:1683–95. 24865794 10.1016/j.pain.2014.05.025 PMC4500524

[64] Gewandter JS McDermott MP McKeown A Hoang K Iwan K Kralovic S Rothstein D Gilron I Katz NP Raja SN Senn S Smith SM Turk DC Dworkin RH Reporting of cross-over clinical trials of analgesic treatments for chronic pain: Analgesic, Anesthetic, and Addiction Clinical Trial Translations, Innovations, Opportunities, and Networks systematic review and recommendations. PAIN 2016;157:2544–51. 27437786 10.1097/j.pain.0000000000000673

[65] Gilron I Drug discovery for neuropathic pain. In: Simpson DM McArthur JC Dworkin RH, editors. Neuropathic pain: mechanisms, diagnosis, and treatment. New York: Oxford University Press, 2012 p. 38–57.

[66] Gilron I Bailey JM Tu D Holden RR Jackson AC Houlden RL Nortriptyline and gabapentin, alone and in combination for neuropathic pain: a double-blind, randomised controlled crossover trial. Lancet 2009;374:1252–61. 19796802 10.1016/S0140-6736(09)61081-3

[67] Gilron I Bailey JM Tu D Holden RR Weaver DF Houlden RL Morphine, gabapentin, or their combination for neuropathic pain. N Engl J Med 2005;352:1324–34. 15800228 10.1056/NEJMoa042580

[68] Gilron I Chaparro LE Tu D Holden RR Milev R Towheed T DuMerton-Shore D Walker S Combination of pregabalin with duloxetine for fibromyalgia: a randomized controlled trial. PAIN 2016;157:1532–40. 26982602 10.1097/j.pain.0000000000000558

[69] Gilron I Jensen TS Dickenson AH Combination pharmacotherapy for management of chronic pain: from bench to bedside. Lancet Neurol 2013;12:1084–95. 24074723 10.1016/S1474-4422(13)70193-5

[70] Gilron I Tu D Holden RR Jackson AC DuMerton-Shore D Combination of morphine with nortriptyline for neuropathic pain. PAIN 2015;156:1440–8. 25749306 10.1097/j.pain.0000000000000149

[71] Granot M Lowenstein L Yarnitsky D Tamir A Zimmer EZ Postcesarean section pain prediction by preoperative experimental pain assessment. Anesthesiology 2003;98:1422–6. 12766652 10.1097/00000542-200306000-00018

[72] Granovsky Y Yarnitsky D Personalized pain medicine: the clinical value of psychophysical assessment of pain modulation profile. Rambam Maimonides Med J 2013;4:e0024. 24228167 10.5041/RMMJ.10131 PMC3820297

[73] Graven-Nielsen T Arendt-Nielsen L Svensson P Jensen TS Quantification of local and referred muscle pain in humans after sequential i.m. injections of hypertonic saline. PAIN 1997;69:111–17. 9060020 10.1016/s0304-3959(96)03243-5

[74] Greenspan JD Craft RM LeResche L rendt-Nielsen L Berkley KJ Fillingim RB Gold MS Holdcroft A Lautenbacher S Mayer EA Mogil JS Murphy AZ Traub RJ Studying sex and gender differences in pain and analgesia: a consensus report. PAIN 2007;132(suppl 1):S26–45. 17964077 10.1016/j.pain.2007.10.014 PMC2823483

[75] Grosen K Fischer IW Olesen AE Drewes AM Can quantitative sensory testing predict responses to analgesic treatment? Eur J Pain 2013;17:1267–80. 23658120 10.1002/j.1532-2149.2013.00330.x

[76] Grosen K Olesen AE Gram M Jonsson T Kamp-Jensen M Andresen T Nielsen C Pozlep G Pfeiffer-Jensen M Morlion B Drewes AM Predictors of opioid efficacy in patients with chronic pain: a prospective multicenter observational cohort study. PLoS One 2017;12:e0171723. 28158269 10.1371/journal.pone.0171723 PMC5291530

[77] Grosen K Vase L Pilegaard HK Pfeiffer-Jensen M Drewes AM Conditioned pain modulation and situational pain catastrophizing as preoperative predictors of pain following chest wall surgery: a prospective observational cohort study. PLoS One 2014;9:e90185. 24587268 10.1371/journal.pone.0090185 PMC3935997

[78] Gustorff B Hoechtl K Sycha T Felouzis E Lehr S Kress HG The effects of remifentanil and gabapentin on hyperalgesia in a new extended inflammatory skin pain model in healthy volunteers. Anesth Analg 2004;98:401–7; table. 14742378 10.1213/01.ANE.0000095150.76735.5D

[79] Hanna M O'Brien C Wilson MC Prolonged-release oxycodone enhances the effects of existing gabapentin therapy in painful diabetic neuropathy patients. Eur J Pain 2008;12:804–13. 18262450 10.1016/j.ejpain.2007.12.010

[80] Harden RN Saracoglu M Connolly S Kirsling A Comstock K Khazey K Gerson T Burns J “Managing” the placebo effect: the single-blind placebo lead-in response in two pain models. Pain Med 2016;17:2305–10. 28025364 10.1093/pm/pnv109

[81] Hatfield I Allison A Flight L Julious SA Dimairo M Adaptive designs undertaken in clinical research: a review of registered clinical trials. Trials 2016;17:150. 26993469 10.1186/s13063-016-1273-9 PMC4799596

[82] Herrmann DN Pannoni V Barbano RL Pennella-Vaughan J Dworkin RH Skin biopsy and quantitative sensory testing do not predict response to lidocaine patch in painful neuropathies. Muscle Nerve 2006;33:42–8. 16228968 10.1002/mus.20419

[83] Hewitt DJ Ho TW Galer B Backonja M Markovitz P Gammaitoni A Michelson D Bolognese J Alon A Rosenberg E Herman G Wang H Impact of responder definition on the enriched enrollment randomized withdrawal trial design for establishing proof of concept in neuropathic pain. PAIN 2011;152:514–21. 21185118 10.1016/j.pain.2010.10.050

[84] Holbech JV Bach FW Finnerup NB Brosen K Jensen TS Sindrup SH Imipramine and pregabalin combination for painful polyneuropathy: a randomized controlled trial. PAIN 2015;156:958–66. 25719617 10.1097/j.pain.0000000000000143

[85] Iannetti GD Zambreanu L Wise RG Buchanan TJ Huggins JP Smart TS Vennart W Tracey I Pharmacological modulation of pain-related brain activity during normal and central sensitization states in humans. Proc Natl Acad Sci U S A 2005;102:18195–200. 16330766 10.1073/pnas.0506624102 PMC1306794

[86] Iavarone L Hoke JF Bottacini M Barnaby R Preston GC First time in human for GV196771: interspecies scaling applied on dose selection. J Clin Pharmacol 1999;39:560–6. 10354959 10.1177/00912709922008164

[87] Ilkjaer S Petersen KL Brennum J Wernberg M Dahl JB Effect of systemic N-methyl-D-aspartate receptor antagonist (ketamine) on primary and secondary hyperalgesia in humans. Br J Anaesth 1996;76:829–34. 8679358 10.1093/bja/76.6.829

[88] Janda AM As-Sanie S Rajala B Tsodikov A Moser SE Clauw DJ Brummett CM Fibromyalgia survey criteria are associated with increased postoperative opioid consumption in women undergoing hysterectomy. Anesthesiology 2015;122:1103–11. 25768860 10.1097/ALN.0000000000000637

[89] Joe HB Kim JY Kwak HJ Oh SE Lee SY Park SY Effect of sex differences in remifentanil requirements for the insertion of a laryngeal mask airway during propofol anesthesia: a prospective randomized trial. Medicine (Baltimore) 2016;95:e5032. 27684878 10.1097/MD.0000000000005032 PMC5265971

[90] Karshikoff B Lekander M Soop A Lindstedt F Ingvar M Kosek E Olgart HC Axelsson J Modality and sex differences in pain sensitivity during human endotoxemia. Brain Behav Immun 2015;46:35–43. 25486090 10.1016/j.bbi.2014.11.014

[91] Katz J Finnerup NB Dworkin RH Clinical trial outcome in neuropathic pain: relationship to study characteristics. Neurology 2008;70:263–72. 17914067 10.1212/01.WNL.0000275528.01263.6c

[92] Katz N Enriched enrollment randomized withdrawal trial designs of analgesics: focus on methodology. Clin J Pain 2009;25:797–807. 19851161 10.1097/AJP.0b013e3181b12dec

[93] Katz NP Mou J Paillard FC Turnbull B Trudeau J Stoker M Predictors of response in patients with postherpetic neuralgia and HIV-associated neuropathy treated with the 8% capsaicin patch (Qutenza). Clin J Pain 2015;31:859–66. 25503598 10.1097/AJP.0000000000000186

[94] Kehlet H Jensen TS Woolf CJ Persistent postsurgical pain: risk factors and prevention. Lancet 2006;367:1618–25. 16698416 10.1016/S0140-6736(06)68700-X

[95] Kelly PJ Sooriyarachchi MR Stallard N Todd S A practical comparison of group-sequential and adaptive designs. J Biopharm Stat 2005;15:719–38. 16022175 10.1081/BIP-200062859

[96] Kharasch ED Regina KJ Blood J Friedel C Methadone pharmacogenetics: CYP2B6 polymorphisms determine plasma concentrations, clearance, and metabolism. Anesthesiology 2015;123:1142–53. 26389554 10.1097/ALN.0000000000000867 PMC4667947

[97] Khoromi S Cui L Nackers L Max MB Morphine, nortriptyline and their combination vs. placebo in patients with chronic lumbar root pain. PAIN 2007;130:66–75. 17182183 10.1016/j.pain.2006.10.029 PMC1974876

[98] Klimas R Witticke D El FS Mikus G Contribution of oxycodone and its metabolites to the overall analgesic effect after oxycodone administration. Expert Opin Drug Metab Toxicol 2013;9:517–28. 23488585 10.1517/17425255.2013.779669

[99] Ko TM Wong CS Wu JY Chen YT Pharmacogenomics for personalized pain medicine. Acta Anaesthesiol Taiwan 2016;54:24–30. 26976339 10.1016/j.aat.2016.02.001

[100] Krarup AL Simren M Funch-Jensen P Hansen MB Hvid-Jensen F Brun J Drewes AM The esophageal multimodal pain model: normal values and degree of sensitization in healthy young male volunteers. Dig Dis Sci 2011;56:1967–75. 21221787 10.1007/s10620-010-1546-1

[101] Laursen RJ Graven-Nielsen T Jensen TS Arendt-Nielsen L Quantification of local and referred pain in humans induced by intramuscular electrical stimulation. Eur J Pain 1997;1:105–13. 15102411 10.1016/s1090-3801(97)90068-9

[102] Lei H Nahum-Shani I Lynch K Oslin D Murphy SA A “SMART” design for building individualized treatment sequences. Annu Rev Clin Psychol 2012;8:21–48. 22224838 10.1146/annurev-clinpsy-032511-143152 PMC3887122

[103] Lemley KJ Hunter SK Bement MK Conditioned pain modulation predicts exercise-induced hypoalgesia in healthy adults. Med Sci Sports Exerc 2015;47:176–84. 24870571 10.1249/MSS.0000000000000381

[104] Lewis JA Statistical principles for clinical trials (ICH E9): an introductory note on an international guideline. Stat Med 1999;18:1903–42. 10440877 10.1002/(sici)1097-0258(19990815)18:15<1903::aid-sim188>3.0.co;2-f

[105] Lin EE Horasek S Agarwal S Wu CL Raja SN Local administration of norepinephrine in the stump evokes dose-dependent pain in amputees. Clin J Pain 2006;22:482–6. 16772803 10.1097/01.ajp.0000202980.51786.ae

[106] Linares OA Daly D Linares AD Stefanovski D Boston RC Personalized oxycodone dosing: using pharmacogenetic testing and clinical pharmacokinetics to reduce toxicity risk and increase effectiveness. Pain Med 2014;15:791–806. 24517173 10.1111/pme.12380

[107] Liu M Max MB Robinovitz E Gracely RH Bennett GJ The human capsaicin model of allodynia and hyperalgesia: sources of variability and methods for reduction. J Pain Symptom Manage 1998;16:10–20. 9707653 10.1016/s0885-3924(98)00026-8

[108] Lotsch J Angst MS The mu-opioid agonist remifentanil attenuates hyperalgesia evoked by blunt and punctuated stimuli with different potency: a pharmacological evaluation of the freeze lesion in humans. PAIN 2003;102:151–61. 12620606 10.1016/s0304-3959(02)00349-4

[109] Magerl W Geldner G Handwerker HO Pain and vascular reflexes in man elicited by prolonged noxious mechano-stimulation. PAIN 1990;43:219–25. 2087332 10.1016/0304-3959(90)91075-T

[110] Mainka T Malewicz NM Baron R Enax-Krumova EK Treede RD Maier C Presence of hyperalgesia predicts analgesic efficacy of topically applied capsaicin 8% in patients with peripheral neuropathic pain. Eur J Pain 2016;20:116–29. 25854794 10.1002/ejp.703

[111] Maixner W Diatchenko L Dubner R Fillingim RB Greenspan JD Knott C Ohrbach R Weir B Slade GD Orofacial pain prospective evaluation and risk assessment study—the OPPERA study. J Pain 2011;12:T4–11. 22074751 10.1016/j.jpain.2011.08.002 PMC3233836

[112] Malfait AM Schnitzer TJ Towards a mechanism-based approach to pain management in osteoarthritis. Nat Rev Rheumatol 2013;9:654–64. 24045707 10.1038/nrrheum.2013.138 PMC4151882

[113] Max MB Single-dose analgesic comparisons. In: Max MB Portenoy RK Laska EM, editors. The design of analgesic clinical trials. New York: Raven Press, 1991 p. 55–95.

[114] Max MB Towards physiologically based treatment of patients with neuropathic pain. PAIN 1990;42:131–7. 1701044 10.1016/0304-3959(90)91156-D

[115] Meloto CB Bortsov AV Bair E Helgeson E Ostrom C Smith SB Dubner R Slade GD Fillingim RB Greenspan JD Ohrbach R Maixner W McLean SA Diatchenko L Modification of COMT-dependent pain sensitivity by psychological stress and sex. PAIN 2016;157:858–67. 26675825 10.1097/j.pain.0000000000000449 PMC4794347

[116] Micheel CM Ball JR Evaluation of biomarkers and surrogate endpoints in chronic disease. Washington: National Academies Press, 2010. 25032382

[117] Miller F Bjornsson M Svensson O Karlsten R Experiences with an adaptive design for a dose-finding study in patients with osteoarthritis. Contemp Clin Trials 2014;37:189–99. 24394343 10.1016/j.cct.2013.12.007

[118] Moore RA Wiffen PJ Eccleston C Derry S Baron R Bell RF Furlan AD Gilron I Haroutounian S Katz NP Lipman AG Morley S Peloso PM Quessy SN Seers K Strassels SA Straube S Systematic review of enriched enrolment, randomised withdrawal trial designs in chronic pain: a new framework for design and reporting. PAIN 2015;156:1382–95. 25985142 10.1097/j.pain.0000000000000088

[119] Muralidharan A Smith MT Pain, analgesia and genetics. J Pharm Pharmacol 2011;63:1387–400. 21988420 10.1111/j.2042-7158.2011.01340.x

[120] Musey PI Jr Linnstaedt SD Platts-Mills TF Miner JR Bortsov AV Safdar B Bijur P Rosenau A Tsze DS Chang AK Dorai S Engel KG Feldman JA Fusaro AM Lee DC Rosenberg M Keefe FJ Peak DA Nam CS Patel RG Fillingim RB McLean SA Gender differences in acute and chronic pain in the emergency department: results of the 2014 Academic Emergency Medicine consensus conference pain section. Acad Emerg Med 2014;21:1421–30. 25422152 10.1111/acem.12529 PMC4390133

[121] Nahum-Shani I Hekler EB Spruijt-Metz D Building health behavior models to guide the development of just-in-time adaptive interventions: a pragmatic framework. Health Psychol 2015;34S:1209–19. 26651462 10.1037/hea0000306 PMC4732268

[122] North RB Kumar K Wallace MS Henderson JM Shipley J Hernandez J Mekel-Bobrov N Jaax KN Spinal cord stimulation versus re-operation in patients with failed back surgery syndrome: an international multicenter randomized controlled trial (EVIDENCE study). Neuromodulation 2011;14:330–5. 21992427 10.1111/j.1525-1403.2011.00371.x

[123] Noto C Pappagallo M Szallasi A NGX-4010, a high-concentration capsaicin dermal patch for lasting relief of peripheral neuropathic pain. Curr Opin Investig Drugs 2009;10:702–10. 19579176

[124] Olesen AE Staahl C Arendt-Nielsen L Drewes AM Different effects of morphine and oxycodone in experimentally evoked hyperalgesia: a human translational study. Br J Clin Pharmacol 2010;70:189–200. 20653672 10.1111/j.1365-2125.2010.03700.x PMC2911549

[125] Olesen SS Graversen C Bouwense SA van GH Wilder-Smith OH Drewes AM Quantitative sensory testing predicts pregabalin efficacy in painful chronic pancreatitis. PLoS One 2013;8:e57963. 23469256 10.1371/journal.pone.0057963 PMC3585877

[126] Oxman AD Guyatt GH A consumer's guide to subgroup analyses. Ann Intern Med 1992;116:78–84. 1530753 10.7326/0003-4819-116-1-78

[127] Paller CJ Campbell CM Edwards RR Dobs AS Sex-based differences in pain perception and treatment. Pain Med 2009;10:289–99. 19207233 10.1111/j.1526-4637.2008.00558.x PMC2745644

[128] Pan PH Coghill R Houle TT Seid MH Lindel WM Parker RL Washburn SA Harris L Eisenach JC Multifactorial preoperative predictors for postcesarean section pain and analgesic requirement. Anesthesiology 2006;104:417–25. 16508387 10.1097/00000542-200603000-00007

[129] Pan PH Tonidandel AM Aschenbrenner CA Houle TT Harris LC Eisenach JC Predicting acute pain after cesarean delivery using three simple questions. Anesthesiology 2013;118:1170–9. 23485992 10.1097/ALN.0b013e31828e156f PMC3951732

[130] Pedersen JL Andersen OK Arendt-Nielsen L Kehlet H Hyperalgesia and temporal summation of pain after heat injury in man. PAIN 1998;74:189–97. 9520233 10.1016/s0304-3959(97)00162-0

[131] Percie du SN Rice AS Improving the translation of analgesic drugs to the clinic: animal models of neuropathic pain. Br J Pharmacol 2014;171:2951–63. 24527763 10.1111/bph.12645 PMC4055199

[132] Petersen KL Meadoff T Press S Peters MM LeComte MD Rowbotham MC Changes in morphine analgesia and side effects during daily subcutaneous administration in healthy volunteers. PAIN 2008;137:395–404. 17977662 10.1016/j.pain.2007.09.019

[133] Petersen KL Rowbotham MC A new human experimental pain model: the heat/capsaicin sensitization model. Neuroreport 1999;10:1511–16. 10380972 10.1097/00001756-199905140-00022

[134] Pocock SJ Hughes MD Lee RJ Statistical problems in the reporting of clinical trials. A survey of three medical journals. N Engl J Med 1987;317:426–32. 3614286 10.1056/NEJM198708133170706

[135] Price N Namdari R Neville J Proctor KJ Kaber S Vest J Fetell M Malamut R Sherrington RP Pimstone SN Goldberg YP Safety and efficacy of a topical sodium channel inhibitor (TV-45070) in patients with postherpetic neuralgia (PHN): a randomized, controlled, proof-of-concept, crossover study, with a subgroup analysis of the Nav1.7 R1150W genotype. Clin J Pain 2017;33:310–18. 28266963 10.1097/AJP.0000000000000408 PMC5348105

[136] Quessy SN Rowbotham MC Placebo response in neuropathic pain trials. PAIN 2008;138:479–83. 18706762 10.1016/j.pain.2008.06.024

[137] Quinlan J Gaydos B Maca J Krams M Barriers and opportunities for implementation of adaptive designs in pharmaceutical product development. Clin Trials 2010;7:167–73. 20338900 10.1177/1740774510361542

[138] Raja SN Campbell JN Meyer RA Evidence for different mechanisms of primary and secondary hyperalgesia following heat injury to the glabrous skin. Brain 1984;107:1179–88. 6509313 10.1093/brain/107.4.1179

[139] Raja SN Jensen TS Predicting postoperative pain based on preoperative pain perception: are we doing better than the weatherman? Anesthesiology 2010;112:1311–12. 20502114 10.1097/ALN.0b013e3181dcd5cc PMC2884220

[140] Riley JL Robinson ME Wise EA Myers CD Fillingim RB Sex differences in the perception of noxious experimental stimuli: a meta-analysis. PAIN 1998;74:181–7. 9520232 10.1016/s0304-3959(97)00199-1

[141] Ruan X Ma L Bumgarner G Is it truly the answer? Personalized oxycodone dosing based on pharmacogenetic testing and the corresponding pharmacokinetics. Pain Med 2016;17:614–15. 26755659 10.1093/pm/pnv082

[142] Salomons TV Moayedi M Erpelding N Davis KD A brief cognitive-behavioural intervention for pain reduces secondary hyperalgesia. PAIN 2014;155:1446–52. 24569149 10.1016/j.pain.2014.02.012

[143] Sang CN Hostetter MP Gracely RH Chappell AS Schoepp DD Lee G Whitcup S Caruso R Max MB AMPA/kainate antagonist LY293558 reduces capsaicin-evoked hyperalgesia but not pain in normal skin in humans. Anesthesiology 1998;89:1060–7. 9821993 10.1097/00000542-199811000-00005

[144] Sang CN Ramadan NM Wallihan RG Chappell AS Freitag FG Smith TR Silberstein SD Johnson KW Phebus LA Bleakman D Ornstein PL Arnold B Tepper SJ Vandenhende F LY293558, a novel AMPA/GluR5 antagonist, is efficacious and well-tolerated in acute migraine. Cephalalgia 2004;24:596–602. 15196302 10.1111/j.1468-2982.2004.00723.x

[145] Schultz DM Webster L Kosek P Dar U Tan Y Sun M Sensor-driven position-adaptive spinal cord stimulation for chronic pain. Pain Physician 2012;15:1–12. 22270733

[146] Senn SS Statistical issues in drug development. West Sussex, United Kingdom: John Wiley & Sons, 2008.

[147] Serra J Duan WR Locke C Sola R Liu W Nothaft W Effects of a T-type calcium channel blocker, ABT-639, on spontaneous activity in C-nociceptors in patients with painful diabetic neuropathy: a randomized controlled trial. PAIN 2015;156:2175–83. 26035253 10.1097/j.pain.0000000000000249

[148] Siegenthaler A Schliessbach J Vuilleumier PH Juni P Zeilhofer HU Arendt-Nielsen L Curatolo M Linking altered central pain processing and genetic polymorphism to drug efficacy in chronic low back pain. BMC Pharmacol Toxicol 2015;16:23. 26376691 10.1186/s40360-015-0023-z PMC4574129

[149] Simone DA Baumann TK Lamotte RH Dose-dependent pain and mechanical hyperalgesia in humans after intradermal injection of capsaicin. PAIN 1989;38:99–107. 2780068 10.1016/0304-3959(89)90079-1

[150] Sindrup SH Finnerup NB Jensen TS Tailored treatment of peripheral neuropathic pain. PAIN 2012;153:1781–2. 22727536 10.1016/j.pain.2012.06.002

[151] Sindrup SH Gram LF Brosen K Eshoj O Mogensen EF The selective serotonin reuptake inhibitor paroxetine is effective in the treatment of diabetic neuropathy symptoms. PAIN 1990;42:135–44. 2147235 10.1016/0304-3959(90)91157-E

[152] Sluka KA Clauw DJ Neurobiology of fibromyalgia and chronic widespread pain. Neuroscience 2016;338:114–29. 27291641 10.1016/j.neuroscience.2016.06.006 PMC5083139

[153] Smith MT Muralidharan A Pharmacogenetics of pain and analgesia. Clin Genet 2012;82:321–30. 22779698 10.1111/j.1399-0004.2012.01936.x

[154] Smith SM Amtmann D Askew RL Gewandter JS Hunsinger M Jensen MP McDermott MP Patel KV Williams M Bacci ED Burke LB Chambers CT Cooper SA Cowan P Desjardins P Etropolski M Farrar JT Gilron I Huang IZ Katz M Kerns RD Kopecky EA Rappaport BA Resnick M Strand V Vanhove GF Veasley C Versavel M Wasan AD Turk DC Dworkin RH Pain intensity rating training: results from an exploratory study of the ACTTION PROTECCT system. PAIN 2016;157:1056–64. 27058680 10.1097/j.pain.0000000000000502

[155] Smith SM Dworkin RH Turk DC Baron R Polydefkis M Tracey I Borsook D Edwards RR Harris RE Wager TD Arendt-Nielsen L Burke LB Carr DB Chappell A Farrar JT Freeman R Gilron I Goli V Haeussler J Jensen T Katz NP Kent J Kopecky EA Lee DA Maixner W Markman JD McArthur JC McDermott MP Parvathenani L Raja SN Rappaport BA Rice AS Rowbotham MC Tobias JK Wasan AD Witter J The potential role of sensory testing, skin biopsy, and functional brain imaging as biomarkers in chronic pain clinical trials: IMMPACT considerations. J Pain 2017;18:757–77. 28254585 10.1016/j.jpain.2017.02.429 PMC5484729

[156] Sprenger C Bingel U Buchel C Treating pain with pain: supraspinal mechanisms of endogenous analgesia elicited by heterotopic noxious conditioning stimulation. PAIN 2011;152:428–39. 21196078 10.1016/j.pain.2010.11.018

[157] Staud R Evidence of involvement of central neural mechanisms in generating fibromyalgia pain. Curr Rheumatol Rep 2002;4:299–305. 12126581 10.1007/s11926-002-0038-5

[158] Sycha T Gustorff B Lehr S Tanew A Eichler HG Schmetterer L A simple pain model for the evaluation of analgesic effects of NSAIDs in healthy subjects. Br J Clin Pharmacol 2003;56:165–72. 12895189 10.1046/j.0306-5251.2003.01869.x PMC1884274

[159] Tchivileva IE Lim PF Smith SB Slade GD Diatchenko L McLean SA Maixner W Effect of catechol-O-methyltransferase polymorphism on response to propranolol therapy in chronic musculoskeletal pain: a randomized, double-blind, placebo-controlled, crossover pilot study. Pharmacogenet Genomics 2010;20:239–48. 20216107 10.1097/FPC.0b013e328337f9ab PMC2876724

[160] Tesfaye S Wilhelm S Lledo A Schacht A Tolle T Bouhassira D Cruccu G Skljarevski V Freynhagen R Duloxetine and pregabalin: high-dose monotherapy or their combination? The “COMBO-DN study”—a multinational, randomized, double-blind, parallel-group study in patients with diabetic peripheral neuropathic pain. PAIN 2013;154:2616–25. 23732189 10.1016/j.pain.2013.05.043

[161] Thall PF Cook JD Dose-finding based on efficacy-toxicity trade-offs. Biometrics 2004;60:684–93. 15339291 10.1111/j.0006-341X.2004.00218.x

[162] Thall PF Cook JD Estey EH Adaptive dose selection using efficacy-toxicity trade-offs: illustrations and practical considerations. J Biopharm Stat 2006;16:623–38. 17037262 10.1080/10543400600860394

[163] Theysohn N Schmid J Icenhour A Mewes C Forsting M Gizewski ER Schedlowski M Elsenbruch S Benson S Are there sex differences in placebo analgesia during visceral pain processing? A fMRI study in healthy subjects. Neurogastroenterol Motil 2014;26:1743–53. 25346054 10.1111/nmo.12454

[164] Thomas JG Bond DS Behavioral response to a just-in-time adaptive intervention (JITAI) to reduce sedentary behavior in obese adults: implications for JITAI optimization. Health Psychol 2015;34S:1261–7. 26651467 10.1037/hea0000304 PMC4681309

[165] Tracey I Neuroimaging mechanisms in pain: from discovery to translation. PAIN 2017;158(suppl 1):S115–22. 28141634 10.1097/j.pain.0000000000000863

[166] Tremblay J Hamet P Genetics of pain, opioids, and opioid responsiveness. Metabolism 2010;59(suppl 1):S5–8. 20837195 10.1016/j.metabol.2010.07.015

[167] Turner JA Deyo RA Loeser JD Von Korff M Fordyce WE The importance of placebo effects in pain treatment and research. JAMA 1994;271:1609–14. 7880221

[168] van AG de Boer MW Groeneveld GJ Hay JL A literature review on the pharmacological sensitivity of human evoked hyperalgesia pain models. Br J Clin Pharmacol 2016;82:903–22. 27203797 10.1111/bcp.13018 PMC5276025

[169] Vardeh D Mannion RJ Woolf CJ Toward a mechanism-based approach to pain diagnosis. J Pain 2016;17:T50–69. 27586831 10.1016/j.jpain.2016.03.001 PMC5012312

[170] Wallace MS Rowbotham MC Katz NP Dworkin RH Dotson RM Galer BS Rauck RL Backonja MM Quessy SN Meisner PD A randomized, double-blind, placebo-controlled trial of a glycine antagonist in neuropathic pain. Neurology 2002;59:1694–700. 12473754 10.1212/01.wnl.0000036273.98213.34

[171] Walsh DA McWilliams DF Mechanisms, impact and management of pain in rheumatoid arthritis. Nat Rev Rheumatol 2014;10:581–92. 24861185 10.1038/nrrheum.2014.64

[172] Wasner G Binder A Baron R Definitions, anatomical localization, and signs and symptoms of neuropathic pain. In: Simpson DM McArthur JC Dworkin RH, editors. Neuropathic pain: mechanisms, diagnosis and treatment. New York: Oxford University Press, 2012 p. 58–75.

[173] Webster LR Belfer I Pharmacogenetics and personalized medicine in pain management. Clin Lab Med 2016;36:493–506. 27514464 10.1016/j.cll.2016.05.007

[174] Wegner A Elsenbruch S Rebernik L Roderigo T Engelbrecht E Jager M Engler H Schedlowski M Benson S Inflammation-induced pain sensitization in men and women: does sex matter in experimental endotoxemia? PAIN 2015;156:1954–64. 26058036 10.1097/j.pain.0000000000000256 PMC4770336

[175] Weissman-Fogel I Granovsky Y Crispel Y Ben-Nun A Best LA Yarnitsky D Granot M Enhanced presurgical pain temporal summation response predicts post-thoracotomy pain intensity during the acute postoperative phase. J PAIN 2009;10:628–36. 19398382 10.1016/j.jpain.2008.12.009

[176] Werner MU Mjobo HN Nielsen PR Rudin A Prediction of postoperative pain: a systematic review of predictive experimental pain studies. Anesthesiology 2010;112:1494–502. 20460988 10.1097/ALN.0b013e3181dcd5a0

[177] Werner MU Petersen KL Rowbotham MC Dahl JB Healthy volunteers can be phenotyped using cutaneous sensitization pain models. PLoS One 2013;8:e62733. 23671631 10.1371/journal.pone.0062733 PMC3650051

[178] Woodcock J Woosley R The FDA critical path initiative and its influence on new drug development. Annu Rev Med 2008;59:1–12. 18186700 10.1146/annurev.med.59.090506.155819

[179] Woolf CJ Bennett GJ Doherty M Dubner R Kidd B Koltzenburg M Lipton R Loeser JD Payne R Torebjork E Towards a mechanism-based classification of pain? PAIN 1998;77:227–9. 9808347 10.1016/S0304-3959(98)00099-2

[180] Wyrick DL Rulison KL Fearnow-Kenney M Milroy JJ Collins LM Moving beyond the treatment package approach to developing behavioral interventions: addressing questions that arose during an application of the Multiphase Optimization Strategy (MOST). Transl Behav Med 2014;4:252–9. 25264465 10.1007/s13142-013-0247-7 PMC4167894

[181] Yamato TP Maher CG Saragiotto BT Shaheed CA Moseley AM Lin CC Koes B McLachlan AJ Comparison of effect sizes between enriched and nonenriched trials of analgesics for chronic musculoskeletal pain: a systematic review. Br J Clin Pharmacol 2017;83:2347–55. 28636752 10.1111/bcp.13350 PMC5651314

[182] Yarnitsky D Conditioned pain modulation (the diffuse noxious inhibitory control-like effect): its relevance for acute and chronic pain states. Curr Opin Anaesthesiol 2010;23:611–15. 20543676 10.1097/ACO.0b013e32833c348b

[183] Yarnitsky D Crispel Y Eisenberg E Granovsky Y Ben-Nun A Sprecher E Best LA Granot M Prediction of chronic post-operative pain: pre-operative DNIC testing identifies patients at risk. PAIN 2008;138:22–8. 18079062 10.1016/j.pain.2007.10.033

[184] Yarnitsky D Granot M Granovsky Y Pain modulation profile and pain therapy: between pro- and antinociception. PAIN 2014;155:663–5. 24269491 10.1016/j.pain.2013.11.005

[185] Yarnitsky D Granot M Nahman-Averbuch H Khamaisi M Granovsky Y Conditioned pain modulation predicts duloxetine efficacy in painful diabetic neuropathy. PAIN 2012;153:1193–8. 22480803 10.1016/j.pain.2012.02.021

[186] Zaslansky R Yarnitsky D Clinical applications of quantitative sensory testing (QST). J Neurol Sci 1998;153:215–38. 9511880 10.1016/s0022-510x(97)00293-1

